# Micro-syringe chip-guided intratumoral administration of lipid nanoparticles for targeted anticancer therapy

**DOI:** 10.1186/s40824-023-00440-4

**Published:** 2023-10-16

**Authors:** Jeongrae Kim, Sunejeong Song, Minjun Gwak, Hanhee Cho, Wan Su Yun, Namcheol Hwang, Jinseong Kim, Jun Seo Lee, Dong-Hwee Kim, Hyuncheol Kim, Seong Ik Jeon, Tae-il Kim, Kwangmeyung Kim

**Affiliations:** 1 College of Pharmacy, Graduate School of Pharmaceutical Sciences, Ewha Woman’s University, Seoul, 03760 Republic of Korea; 2 KU-KIST Graduate School of Converging Science and Technology, Korea University, Seoul, 02841 Republic of Korea; 3 School of Chemical Engineering, Sungkyunkwan University (SKKU), Suwon, 16419 Republic of Korea; 4 Department of Chemical and Biomolecular Engineering, Sogang University, Seoul, 04107 Republic of Korea

**Keywords:** Micro-syringe, Drug delivery system, Intratumoral administration, Lipid nanoparticle, Anticancer therapy

## Abstract

**Background:**

Nano-sized drug delivery system has been widely studied as a potential technique to promote tumor-specific delivery of anticancer drugs due to its passive targeting property, but resulting in very restricted improvements in its systemic administration so far. There is a requirement for a different approach that dramatically increases the targeting efficiency of therapeutic agents at targeted tumor tissues.

**Methods:**

To improve the tumor-specific accumulation of anticancer drugs and minimize their undesirable toxicity to normal tissues, a tumor-implantable micro-syringe chip (MSC) with a drug reservoir is fabricated. As a clinically established delivery system, six liposome nanoparticles (LNPs) with different compositions and surface chemistry are prepared and their physicochemical properties and cellular uptake are examined in vitro. Subsequently, MSC-guided intratumoral administration is studied to identify the most appropriate for the higher tumor targeting efficacy with a uniform intratumoral distribution. For efficient cancer treatment, pro-apoptotic anticancer prodrugs (SMAC-P-FRRG-DOX) are encapsulated to the optimal LNPs (SMAC-P-FRRG-DOX encapsulating LNPs; ApoLNPs), then the ApoLNPs are loaded into the 1 μL-volume drug reservoir of MSC to be delivered intratumorally for 9 h. The tumor accumulation and therapeutic effect of ApoLNPs administered via MSC guidance are evaluated and compared to those of intravenous and intratumoral administration of ApoLNP in 4T1 tumor-bearing mice.

**Results:**

MSC is precisely fabricated to have a 0.5 × 4.5 mm needle and 1 μL-volume drug reservoir to achieve the uniform intratumoral distribution of LNPs in targeted tumor tissues. Six liposome nanoparticles with different compositions of 1-palmitoyl-2-oleoyl-glycero-3-phosphocholine (PC), 1,2-dioleoyl-sn-glycero-3-phospho-L-serine (PS), 1,2-dioleoyl-3-trimethylammonium-propane (DOTAP), and 1,2-distearoyl-sn-glycero-3-phosphoethanolamine-N-[methoxy (polyethylene glycol)_2000_] (PEG_2000_-DSPE) are prepared with average sizes of 100–120 nm and loaded into the 1 μL-volume drug reservoir in MSC. Importantly negatively charged 10 mol% of PS-containing LNPs are very slowly infused into the tumor tissue through the micro-syringe of the MSC over 6 h. The intratumoral targeting efficiency of MSC guidance is 93.5%, effectively assisting the homogeneous diffusion of LNPs throughout the tumor tissue at 3.8- and 2.7-fold higher concentrations compared to the intravenous and intratumoral administrations of LNPs, respectively. Among the six LNP candidates 10 mol% of PS-containing LNPs are finally selected for preparing pro-apoptotic SMAC-P-FRRG-DOX anticancer prodrug-encapsulated LNPs (ApoLNPs) due to their moderate endocytosis rate high tumor accumulation and homogenous intratumoral distribution. The ApoLNPs show a high therapeutic effect specifically to cathepsin B-overexpressing cancer cells with 6.6 μM of IC_50_ value while its IC_50_ against normal cells is 230.7 μM. The MSC-guided administration of ApoLNPs efficiently inhibits tumor growth wherein the size of the tumor is 4.7- and 2.2-fold smaller than those treated with saline and intratumoral ApoLNP without MSC, respectively. Moreover, the ApoLNPs remarkably reduce the inhibitor of apoptosis proteins (IAPs) level in tumor tissues confirming their efficacy even in cancers with high drug resistance.

**Conclusion:**

The MSC-guided administration of LNPs greatly enhances the therapeutic efficiency of anticancer drugs via the slow diffusion mechanism through micro-syringe to tumor tissues for 6 h, whereas they bypass most hurdles of systemic delivery including hepatic metabolism, rapid renal clearance, and interaction with blood components or other normal tissues, resulting in the minimum toxicity to normal tissues. The negatively charged ApoLNPs with cancer cell-specific pro-apoptotic prodrug (SMAC-P-FRRG-DOX) show the highest tumor-targeting efficacy when they are treated with the MSC guidance, compared to their intravenous or intratumoral administration in 4T1 tumor-bearing mice. The MSC-guided administration of anticancer drug-encapsulated LNPs is expected to be a potent platform system that facilitates overcoming the limitations of systemic drug administration with low delivery efficiency and serious side effects.

**Graphical Abstract:**

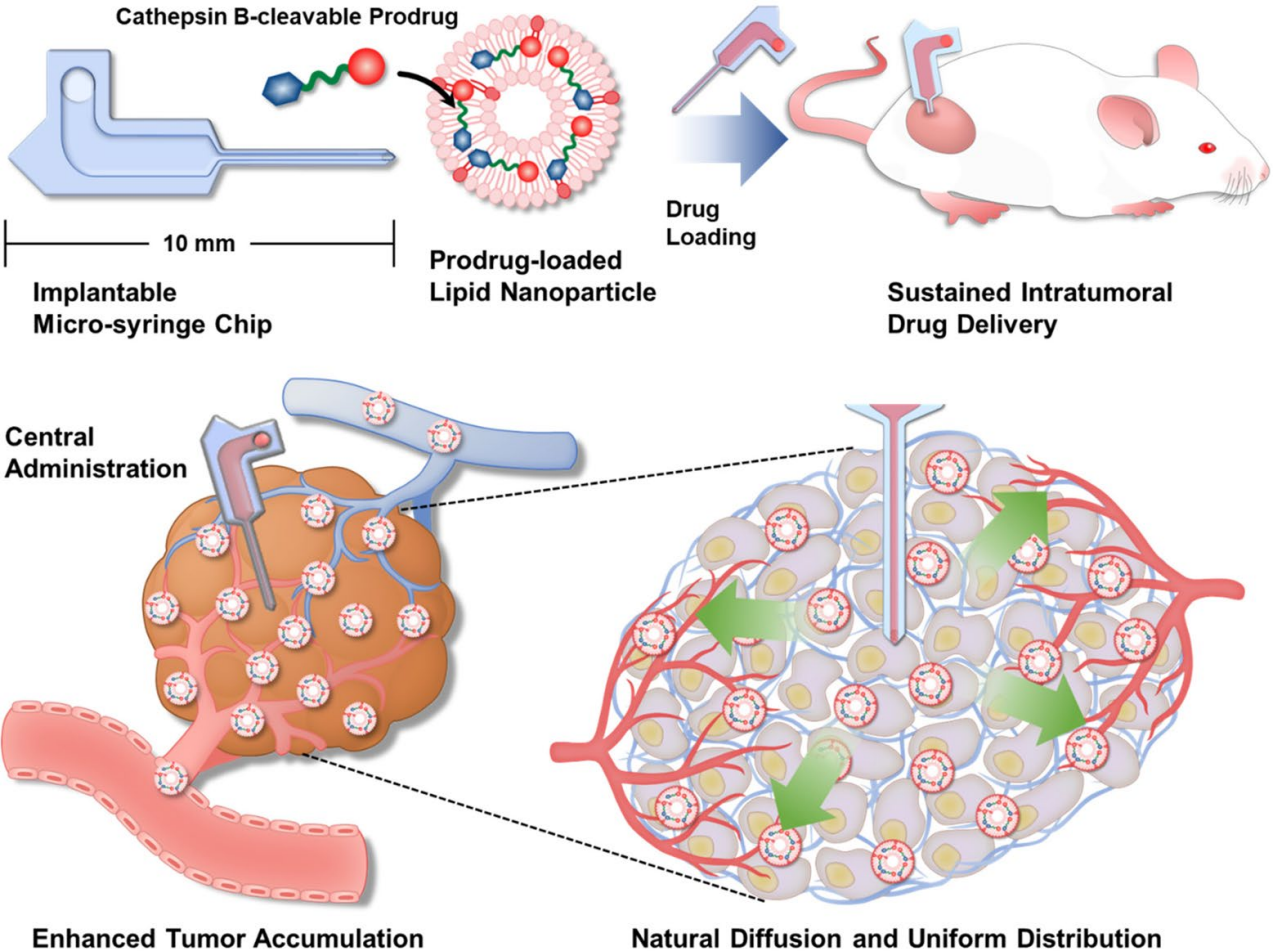

**Supplementary Information:**

The online version contains supplementary material available at 10.1186/s40824-023-00440-4.

## Background

Nano-sized drug delivery systems (nanoDDSs) have been actively employed for cancer treatment as they have been perceived as a new paradigm over conventional anticancer drugs [1]. It has been extensively reported that nanoDDSs exhibit better pharmacokinetic properties compared to anticancer drugs due to their high stability in physiological conditions and prolonged blood circulation [2–4]. In addition, the intravenously administered nanoDDSs tend to specifically accumulate inside the tumor via the enhanced permeability and retention (EPR) effect, resulting in enhanced therapeutic outcomes [5, 6]. The tumor-specific accumulation of nanoDDS also mitigates the side effects of anticancer drugs caused by undesirable delivery to normal tissues. However, nanoDDSs have not been successful in validating their tumor-specific efficacy in the clinical phase so far [7–10]. When nanoDDSs are systemically administered, they often suffer from several obstructions such as interactions with blood components and entrapment to the reticuloendothelial system (RES), which greatly disturb their passive tumor targeting and make it hard to predict their biodistribution [11–14]. The metabolism and excretion by the liver and kidney and the non-specific distribution to normal tissues are also unavoidable factors that decrease the tumor delivery efficiency of nanoDDSs. The actual delivery efficiency of systemic nanoDDS administration was reported not to exceed 1–2%, showing a limited advancement from conventional anticancer drugs without nanocarrier, only to aggravate further problems of carrier-derived toxicity and immunogenicity [15–18]. The lower delivery efficiency and off-target localization of nanoDDSs are still unsolved problems in cancer treatment.

Direct intratumoral administration of nanoDDSs can be an alternative to achieve the desired drug biodistribution at targeted tumor tissues [19–21]. Through intratumoral administration, nanoDDSs can bypass most hurdles of systemic delivery, including hepatic metabolism, rapid renal clearance, and interaction with blood components or other normal tissues [22]. Allowing the nanoDDSs to be specifically localized inside the tumors, it dramatically enhances their therapeutic effect while alleviating their side toxicity. Notably, nanoDDSs are more beneficial than small molecular anticancer drugs for intratumoral delivery as it attenuates the immediate diffusion of anticancer drugs through the extracellular matrix and their wash-out from the lesional tissue [23]. To enlarge the therapeutic effect of intratumoral nanoDDS administration, it is important to infuse nanoDDSs at a prolonged and controlled rate [24, 25]. When nanoDDSs are intratumorally administered at once, an excessive amount of them is unavoidably leaked out to normal tissues, lymphatic drainage, or blood circulation due to the insufficient time to be uniformly dispersed throughout the tumor tissue and endocytosed by cancer cells.

Recently, microneedles (MNs) have been explored for the direct intratumoral delivery of anticancer drugs [26–28]. MNs are a minimally invasive and highly localized method for cancer treatment, which assist in the subcutaneous infusion of anticancer drugs at a precisely controlled release rate [29, 30]. Therefore, MN-mediated drug delivery can efficiently prevent the side effects due to unwanted drug distribution to normal organs. They are universally applicable to various types of anticancer drugs including chemodrugs, proteins, nucleotides, and nanoDDSs. However, MNs have some problems in their application to deep tumor tissues since they were originally developed for transdermal drug delivery rather than intratumoral administration [31]. The needle length of MNs generally does not exceed 1–1.5 mm considering the thickness of the epidermis and dermis, thereby presenting a short penetration depth and limited delivery efficiency to deep tumor tissues [32, 33]. The hypodermic drug administration by MNs inevitably confronts drug diffusivity and skin irritation issues [34, 35]. Moreover, the drug loading capacity of MNs is extremely small since drugs were typically embedded in the thin polymer coat on the needles [36]. Poor drug encapsulation is a persistent drawback of MNs, even using hollow- or dissolving-type needles. The complexity of the drug-embedded MN fabrication process is an additional hurdle in its application as the procedures are accompanied by at least four steps [37].

**Scheme 1:**
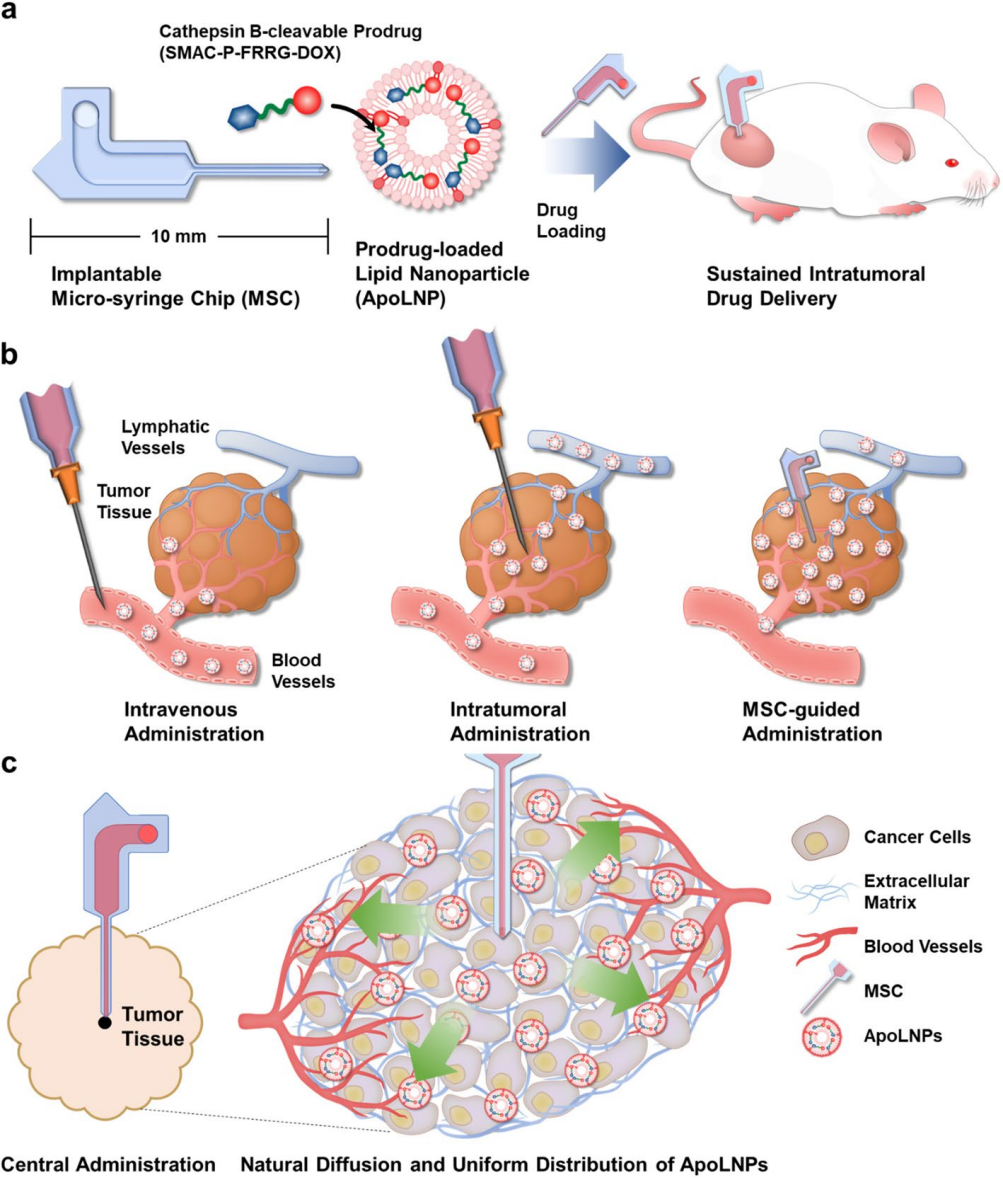
Schematic illustration for MSC-guided sustained intratumoral drug delivery (**a**) The intratumorally implantable MSC composed of a drug-infusing needle and a reservoir was fabricated, and the cathepsin B-cleavable pro-apoptotic prodrug (SMAC-P-FRRG-DOX)-encapsulating LNPs (ApoLNPs) were loaded in the reservoir of MSC. The MSC-guided intratumoral administration allows ApoLNPs to be directly infused into deep tumor tissues in a persistent manner. **b** Compared to the intravenous or instant intratumoral administration, the MSC-guided administration was expected to secure more tumor-exclusive accumulation and uniform intratumoral distribution of drugs by diminishing their undesirable delivery to normal tissues or rapid leakage from tumors. **c** ApoLNPs would be slowly released at the center of tumor tissues through the MSC guidance and evenly distributed to entire tissues via their gradual diffusion mechanism

Micro-syringe-type devices, which consist of longer drug-administering needles and reservoirs, are rather more advantageous than MNs for deep tissue administration as they can infuse nanoDDSs into the center of tumor tissues through their microchannel. Herein, a new intratumoral administration system composed of an implantable micro-syringe chip (MSC) and pro-apoptotic prodrug-encapsulated lipid nanoparticles (ApoLNPs) was proposed to enhance the anticancer efficacy and reduce the side effect (Scheme 1a). The MSC was designed to be directly applied to deep tumor tissues and let the solutions naturally diffuse from its drug reservoir to the deep site of tumor tissues through its needle at a delayed diffusion mechanism, securing their exclusive and sufficient tumoral accumulation. Additionally, it was attempted to discover the most efficient lipid nanoparticles (LNPs) which can smoothly diffuse into tumor tissues through the MSC guidance. Six model LNPs with different surface chemistries and zeta potentials were prepared and their cellular uptake and cytotoxicity were analyzed to optimize the physicochemical properties of LNPs that can be diffused into deep tumor tissues. Thereafter, the MSC-guided intratumoral administration of six LNPs was carried out against 4T1 tumor-bearing mice, and the distributions of infused LNPs inside tumor tissues and other normal organs were observed. After optimizing the composition of LNPs, a cathepsin B-cleavable prodrug (SMAC-P-FRRG-DOX) consisting of both second mitochondria-derived activator of caspases mimetic peptide (SMAC-P) and chemodrug (doxorubicin; DOX) was encapsulated into LNPs to form ApoLNPs that can induce potent apoptosis of targeted tumor cells in synergy of SMAC-P and DOX. The fabricated ApoLNPs were assessed for their size, cathepsin B-specific activatability, and cytotoxicity against normal and cancer cells, then they were intratumorally administered via MSC guidance to monitor their tumor accumulation efficiency compared to its intravenous or direct intratumoral administration without MSC (Scheme 1b). The MSC-guided ApoLNP administration was expected to achieve higher intratumoral accumulation than other intravenous and intratumoral administration routes. Also, the MSC guidance let ApoLNPs be released at the center of tumor tissues so that ApoLNPs could uniformly diffuse throughout the whole tumor tissues (Scheme 1c). Subsequently, the therapeutic effect of MSC-guided administration of ApoLNPs was evaluated against 4T1 tumor-bearing mice, and their property to alleviate the drug resistance of tumors was evaluated.

## Methods

### Regents

SU-8 epoxy photoresist (SU-8 100) was purchased from MicroChem Inc. (Newton, MA, USA) and polysiloxane acrylate (PSA) precursor (ST1010s) was obtained from MCNet Inc. (Gwangju, Republic of Korea). 1-palmitoyl-2-oleoyl-glycero-3-phosphocholine (PC), 1,2-distearoyl-sn-glycero-3-phosphoethanolamine-N-[methoxy (polyethylene glycol)_2000_] (PEG_2000_-DSPE), 1,2-dioleoyl-sn-glycero-3-phospho-L-serine (PS), 1,2-dioleoyl-3-trimethylammonium-propane (DOTAP), and 1-palmitoyl-2-oleoyl-sn-glycero-3-phosphoethanolamine (PE) were purchased from Avanti Polar Lipids, Inc. (Alabaster, AL, USA). Polydimethylsiloxane (PDMS, Sylgard 184), cholesterol (CHOL), N,N-diisopropylethylamine (DIPEA), 1-ethyl-3-(3-dimethylaminopropyl) carbodiimide (EDC), N-hydroxysuccinimide (NHS), chloroform, anhydrous N,N-dimethylformamide (DMF), and dimethyl sulfoxide (DMSO) were obtained from Sigma-Aldrich (St. Louis, MO, USA), and Flamma® Fluor 648-NHS ester dye (Flamma 648) was available from BioActs (Incheon, Republic of Korea). Doxorubicin hydrochloride (DOX) and *acetyl-Ala-Val-Pro-Ile-Ala-Gln-Phe-Arg-Arg-Gly* (Ac-AVPIAQ-FRRG; SMAC-P-FRRG) were obtained from FutureChem (Seoul, Republic of Korea), and recombinant human cathepsin B protein was purchased from R&D Systems (Minneapolis, MN, USA).

### Instruments

The size, zeta potential, and morphology of LNPs were analyzed via dynamic light scattering (DLS; Zetasizer Nano ZS, Malvern Instruments, Worcestershire, UK) and cryogenic transmission electron microscopy (CryoTEM; Tecnai F20, FEI, Eindhoven, Netherlands). A confocal laser scanning microscope (CLSM; Leica TCS SP8, Leica Microsystems GmbH; Wetzlar, Germany) was used to take the in vitro cellular and ex vivo tissue fluorescence images. The in vitro cell viability was assessed using a microplate reader (VersaMax™, Molecular Devices Corp., Palo Alto, CA, USA). To confirm the synthesis and cathepsin B-specific cleavage of SMAC-P-FRRG-DOX, high-performance liquid chromatography equipped with a mass spectroscopy system (HPLC/LC–MS; 1260 Infinity II LC system, Agilent Technologies, Santa Clara, CA, USA) was utilized. The drug release profile was assessed using a UV–vis spectrophotometer (Agilent Cary 300; Agilent Technologies, Santa Clara, CA, USA). The in vivo fluorescence images of mice were observed via in vivo imaging system (IVIS; IVIS® Lumina Series III, PerkinElmer, Waltham, MA, USA), equipped at Ewha Drug Development Research Core Center.

### Fabrication of implantable MSC

The bottom (microchannel with a reservoir) and lid (with inlet and outlet holes) parts of MSC were independently fabricated via photolithography. For the fabrication of the bottom part, a negative photoresist SU-8 100 was spun on a clean silicon wafer at 3000 rpm to derive the depth of the reservoir to be about 40 μm. As a general procedure for SU-8, soft bake at 65 °C, UV exposure, and post-exposure bake at 95 °C were applied in orders. After the dissolution of the photoresist using SU-8 developer, the patterns of the bottom could be obtained. The lid patterns were also generated by the same procedure for both inlet and outlets. With these SU-8 master molds, PDMS replicas were fabricated using the precursor and curing agent mixed in a 10:1 ratio. Both PDMS replicas with bottom and lid patterns were treated by UVO and bonded together by applying a high temperature of 150 °C for 1 h. After the PSA precursor was filled between these two bonded PDMS, it was pre-cured by UV irradiation (250–400 nm, 60 mJ/cm^2^) and its mechanical demolding was carried out. The uncured residual PSA precursor was subsequently removed using isopropanol. The patterned PSA parts in the shape of the lid and bottom were precisely aligned using a mask aligner, followed by their adherence with the uncured PSA as an adhesive. The whole part was further exposed to UV (10 mJ/cm^2^, 12 h) to obtain the fully cured MSC.

### Preparation of LNP candidates

To endow the fluorescence visibility to LNPs, Flamma 648-conjugated PE (Flamma 648-PE) was synthesized and included in LNPs. Briefly, Flamma 648 (0.8 mg, 1.0 μmol) and PE (0.7 mg, 1.0 μmol) were dissolved in 0.2 mL anhydrous DMF and added with DIPEA (0.2 μL, 1.1 nmol). The mixture was allowed to react between the NHS esters and amines to form ester bonds. After a 12 h reaction, the resulting solution was subjected to column chromatography using a Sep-Pak C18 cartridge (Waters Corp., Milford, MA, USA) to separate Flamma 648-PE. Subsequently, six LNPs with different lipid compositions were formed using the film-casting method. In general, a 30.0 mg lipid mixture was dissolved in 1 mL chloroform and poured into a 25 mL one-neck round-bottom flask. With a steady rotation, the solvent was slowly evaporated for 15 min under reduced pressure to produce a uniform lipid film on the inner wall of the flask. The lipid film was hydrated with 1.0 mL triple distilled water (3’DW) and agitated with a probe-type sonicator for 15 min to obtain LNPs. The molar ratios of lipid mixtures for LNP candidates were fixed to: PC (PC:CHOL = 90:10), 10PEG (PC:PEG_2000_-DSPE:CHOL = 81:9:10), 10PS (PC:PS:CHOL = 81:9:10), 20PS (PC:PS:CHOL = 72:18:10), 10DOTAP (PC:DOTAP:CHOL = 81:9:10), and 20DOTAP (PC:DOTAP:CHOL = 72:18:10), and all LNPs were additionally contained with 0.5% Flamma 648-PE. The formulated LNPs were diluted to 1 mg/mL in PBS and evaluated with DLS and CyroTEM. The LNP solutions were further assessed for their dispersion stability in PBS for up to 6 days.

### In vitro cellular uptake and viability assays of LNP candidates

For the in vitro cellular assay, rat heart myoblast cells (H9C2) and mouse breast cancer cells (4T1) were purchased from American Type Culture Collection (ATCC; Manassas, VA, USA). To examine the time-dependent cellular uptake of LNP candidates, 5 × 10^4^ 4T1 cells were seeded in confocal dishes and treated with six LNPs of 0.1 mg/mL concentration respectively. At 0, 1, 6, and 24 h after the LNP treatment, the cells were washed three times with PBS and fixed with a 4% formaldehyde solution for 15 min. Subsequently, they were stained with 4',6-diamidino-2-phenylindole (DAPI) for 15 min, washed three times with PBS again, and the stained cells were observed via CLSM. For in vitro cytotoxicity assay of LNPs, 5 × 10^3^ 4T1 cells were dispensed in each well of a 96-well plate and cultured for 24 h. The cells were treated with six LNPs at various concentrations from 0.01 to 200 μM for 24 h, washed three times with PBS, added with 5% cell counting kit-8 (CCK-8; Vitascientific, Beltsville, MD, USA) solution, then their light absorbance at 450 nm wavelength was measured using a microplate reader.

### In vivo MSC-guided intratumoral delivery of LNP candidates to tumor-bearing mice

For the in vivo assessment, five-week-old female BALB/c mice were obtained from NARA Biotech (Seoul, Republic of Korea). All experimental mice were fed and bred under the specific-pathogen-free (SPF) facilities at the College of Pharmacy, Graduate School of Pharmaceutical Sciences, Ewha Womans University. All in vivo experiments were conducted in compliance with the guidelines and regulations of the institutional animal care and use committee (IACUC) at Ewha Womans University (approval no. EWHA IACUC 22–073-2). To prepare the 4T1 tumor-bearing model, 1 × 10^6^ 4T1 cells were inoculated to the left thighs of five-week-old female mice (*n* = 3). When the tumor volume reached ~ 200 mm^3^, six LNP solutions (30 mg/mL, 1 μL) and free Flamma 648 solution (0.15 mg/mL, 1 μL) were separately loaded in the MSCs and directly implanted in the center of the tumor tissues after pre-punching a hole with a 26 G medical needle. The LNPs and Flamma 648 dye were let diffused into tumor tissues through MSC guidance for 9 h, and the IVIS images of recipient mice were obtained at 0, 1, 3, 6, and 9 h of the administration. The fluorescence images of MSCs before and after the administration were also taken and their intensities were quantified to calculate the release amount. After 9 h of administration, mice were sacrificed and their tumor tissues were isolated for further evaluation. The lower hemispheres of tumor tissues were embedded in the optimal cutting temperature (OCT) compound (Tissue-Tek®, Sakura Finetek, Torrance, CA, USA) and cryo-sliced to obtain 5 μm-thick sections at three different parts of the tissues. The sections were stained with DAPI for 15 min, washed three times with PBS, and the whole section was observed via CLSM to determine the intratumoral distribution of LNPs. The remaining upper parts of the tumor tissues were put into the co-solvent of 0.9 mL DMSO and 0.1 mL methanol and ground using a homogenizer. The ground suspensions were centrifuged at 15,000 xg for 15 min then 0.5 mL supernatants were taken to be diluted with 0.5 mL chloroform. The solutions were centrifuged again at 15,000 xg for 15 min and the supernatants were analyzed via HPLC to quantify the tumor tissue-remaining LNP or dye amounts.

### Synthesis of SMAC-P-FRRG-DOXs

SMAC-P-FRRG (10.0 mg, 8.2 μmol) was placed into a 3 mL one-neck round-bottom flask equipped with a magnetic stirrer bar and dissolved in 0.5 mL DMF. The solution was added with EDC (2.4 mg, 12.4 μmol) and NHS (1.4 mg, 12.4 μmol) to activate carboxylic acid groups for 1 h. DOX (9.6 mg, 16.5 μmol) and DIPEA (0.1 μL, 0.8 μmol) were successively put into the solution and the amidation reaction between SMAC-P-FRRG and DOX was carried out for 12 h. Upon completing the reaction, the resulting solution was subjected to column chromatography using a C18 column to obtain SMAC-P-FRRG-DOX. The synthesized SMAC-P-FRRG-DOX was analyzed via HPLC equipped with a C18 column and LC–MS. Further, SMAC-P-FRRG-DOX (0.04 mg, 20 nmol) was dissolved in 0.2 mL MES buffer and incubated together with cathepsin B (10 μg/mL) to determine its cathepsin B-responsive cleavage. After 24 h, the resulting mixture was analyzed via HPLC and LC–MS.

### Preparation and characterization of ApoLNPs

To encapsulate the SMAC-P-FRRG-DOX inside the LNP, a SMAC-P-FRRG-DOX solution in ethanol (5 mg/mL, 0.6 mL) and a lipid mixture (10PS; PC:PS:CHOL = 81:9:10) in chloroform (30 mg/mL, 1.0 mL) were separately prepared and put together into a 25 mL one-neck round-bottom flask. The mixture was evaporated, hydrated with 1.0 mL 3’DW, and sonicated to formulate ApoLNPs through the film-casting method. The formed ApoLNPs were diluted to 1 mg/mL in PBS and analyzed via DLS and CryoTEM. Thereafter, 1 mL ApoLNP solution (15 mg/mL) was placed into a membrane bag with 12–14 kDa molecular weight cut off (MWCO) and dialyzed with 10 mL PBS for 10 days, and the diluent was assessed with UV–vis spectroscopy at 480 nm wavelength to determine the release profile of SMAC-P-FRRG-DOX from the LNP.

### In vitro cellular uptake and viability assays of ApoLNPs

Before evaluating the cellular uptake and cytotoxicity of ApoLNPs, the western blot assay of 4T1 and H9C2 cells was carried out for comparing their cathepsin B expression levels. To confirm the endocytosis behavior of ApoLNP, 5 × 10^4^ 4T1 cells were cultured in confocal dishes for 24 h and treated with 5 μM (based on SMAC-P-FRRG-DOX content) ApoLNPs for 1, 6, and 24 h. After fixing the cells with 4% formaldehyde solution for 15 min, the cells were washed three times with PBS, stained with DAPI for 15 min, and repeatedly washed with PBS. The time-dependent changes in the intracellular distribution of Flamma 648-PE and DOX were monitored via CLSM. The cytotoxicity test of ApoLNP was conducted against both 4T1 and H9C1 cells. In each well of 96-well plates, 5 × 10^3^ of 4T1 or H9C2 cells were incubated for 24 h, and treated with DOXs or ApoLNPs at concentrations of 0.01–200 μM for another 24 h. Further added with 5% CCK-8 solution for 20 min, the cells were examined for their 450 nm light absorbance using the microplate reader. The western blot of DOX- or ApoLNP-treated cells was also performed to compare their inhibitor of apoptosis proteins (IAP) expression levels.

### In vivo MSC-guided intratumoral delivery of ApoLNPs to tumor-bearing mice

1 × 10^6^ 4T1 cells were subcutaneously administered into the left thighs of five-week-old female mice to prepare tumor-bearing models (*n* = 3). As their tumor volumes grew to ~ 200 mm^3^, ApoLNPs (0.15 mg/kg, based on DOX content) were administered to the tumor-bearing mice intravenously, intratumorally with, or without MSC guidance. Free DOXs (0.15 mg/kg, based on DOX content) and SMAC-P-FRRG-DOXs (0.15 mg/kg, based on DOX content) were also intratumorally administered with the MSC guidance. The intravenous and intratumoral administrations without MSC were performed at once, and the MSC-guided administration was carried out for 6 h. The in vivo biodistribution and tumor accumulation of free DOXs, SMAC-P-FRRG-DOXs, and ApoLNPs were monitored via the IVIS for up to 48 h. All recipient mice were sacrificed and their tumor tissues were collected. The ex vivo fluorescence images of excised tumors were obtained using the IVIS, then the tumor tissues were cryo-sectioned at 5 μm thickness with OCT compound. The sections were stained with DAPI for 15 min, washed three times with PBS, and observed via CLSM to confirm the intratumoral distribution of ApoLNPs. To investigate the pharmacokinetic profiles of ApoLNPs with different administration routes, ApoLNPs were administered intravenously (0.15 or 5 mg/kg, based on DOX content), intratumorally (0.15 mg/kg, based on DOX content), and MSC-guided intratumorally (0.15 mg/kg, based on DOX content) to 4T1 tumor-bearing mice, and the whole blood of recipient mice was taken at 20 and 40 min, 1, 3, 6, 24, and 48 h post-administration to quantify the blood plasma concentrations of ApoLNPs via HPLC analysis. The normal organs of recipient mice were also collected to observe the time-dependent off-target distribution of ApoLNPs through ex vivo IVIS imaging.

### In vivo therapeutic efficacy of MSC-guided administered ApoLNPs against tumor-bearing mice

The repeated treatment of ApoLNPs was conducted on tumor-bearing mice via intravenous, intratumoral, and MSC-guided intratumoral approaches to compare their therapeutic efficacy. The tumor-bearing mouse models were prepared by inoculating 1 × 10^6^ 4T1 cells to the left thighs of five-week-old female mice (*n* = 3). When tumor volumes increased to ~ 100 mm^3^, ApoLNPs (0.15 mg/kg, based on DOX content) were administered through three different routes once every 3 days, for a total of 3 times. During the repeated administration, the IVIS images of the mice were obtained. The body weights and tumor volumes of all experimental groups were measured once per 2 days for up to 12 days. Upon finishing the monitoring, all mice were sacrificed and their tumor tissues and normal organs including the hearts, livers, lungs, spleens, and kidneys were extracted. The tumor tissues and organs were cryo-sectioned using the OCT compound and underwent hematoxylin and eosin (H&E) staining for their histologic assay. Tumor tissues were further stained with terminal deoxynucleotidyl transferase dUTP nick end labeling (TUNEL) and DAPI to determine the degree of tumor apoptosis via the CLSM image, and their western blot assay was performed to confirm their IAP levels.

### Statistics

The student’s t-test was used for the comparison of two experimental groups, and the statistical analysis among three or more experimental groups was conducted via a one-way analysis of variance (ANOVA) test. The statistical significance between mean values was determined through the Tukey–Kramer method. All data in this study were denoted as average ± standard deviation (SD) after at least three repetitions and their statistical significance was indicated in the graphs with asterisks (* *p* < 0.05, ** *p* < 0.01, *** *p* < 0.001, **** *p* < 0.0001). p-values lower than 0.05 were considered statistically valid.

## Results

### Characterizations of implantable MSC and LNP candidates

**Fig. 1:**
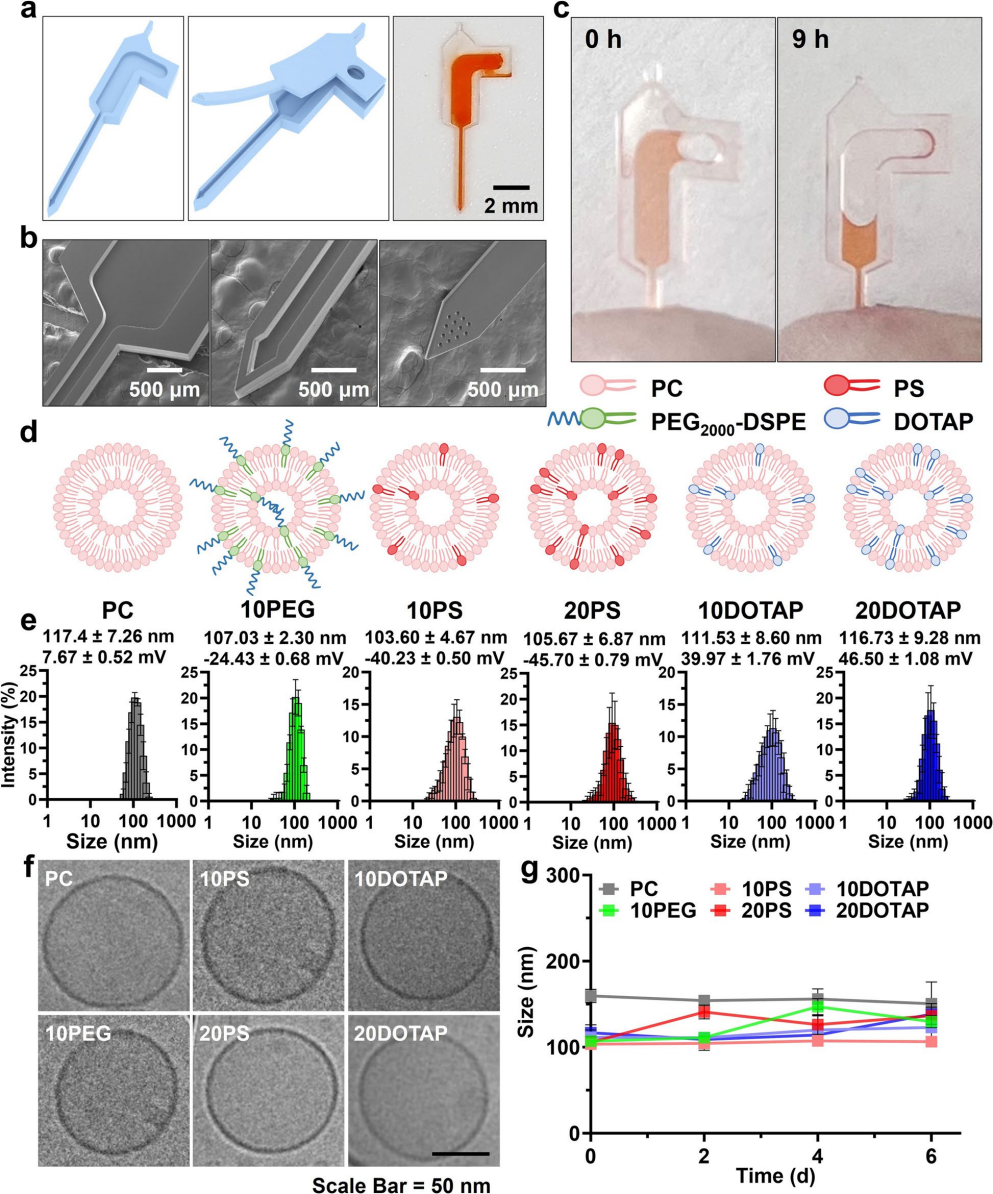
Characterization of fabricated MSC and LNP candidates (**a**) The lid and reservoir-including bottom parts of MSC were separately fabricated and sealed together using the PSA precursor. The digital image of the drug-loaded MSC showed that the drug solution was stably charged inside the reservoir of the MSC. **b** The TEM images of the MSC showed its precise structure with an accurately shaped reservoir and needle, and drug-infusing pinholes on the tip of the needle. **c** The MSC was smoothly implanted directly into the tumor tissues, slowly infusing drugs without any leakage. **d** Six LNP candidates with different compositions and surface properties were prepared and **e** their hydrodynamic diameters and zeta potentials were measured. **f** The cryoTEM images of LNPs exhibited their uniform circular shapes and hollow structures. **g** The changes in sizes of LNPs over time were measured via DLS, demonstrating their high dispersion stability for 6 days

To achieve the sustained and uniform intratumoral administration of LNPs, an implantable drug-infusing MSC was precisely fabricated via the photolithography method (Fig. S1). The reservoir-containing bottom and lid parts of the MSC were separately manufactured by photo-crosslinking PSA, and then attached to each other using the PSA precursor (Fig. 1a). The size of the fabricated MSC was 10.0 × 3.7 × 0.1 mm (height x width x thickness) and 1 μL of drug solution could be loaded stably in the drug reservoir of the MSC through a single inlet hole (500 μm in diameter) on the lid. When observed with TEM, the depth of the reservoir was accurately controlled to ~ 40 μm for securing a constant drug loading amount (Fig. 1b). The outer and inner widths of the needle part were uniformly measured as 540 and 200 μm, respectively. Thirteen micro-holes of 40 μm in diameter were arrayed at the tip of the needle, which enabled the gradual release of drug solutions out of the reservoir. Since the MSC was produced using the biocompatible PSA, it was harmless when implanted in bodies for a long time [38]. Moreover, its needle of over 5 mm in length enabled the deep tumor tissue delivery of LNPs. The MSC was directly implantable into tumor tissues after punching a pinhole onto the skin with 26 G stainless needles, smoothly discharging the loaded drug solution without any significant leakage or reflux (Fig. 1c). The MSC was tough and flex enough to endure the possible needle breakage, and it did not cause serious bleeding from the implant area during its 9 h administration.

The selection of the most desirable LNPs for MSC-guided administration was essential since LNPs would considerably affect the drug delivery efficiency in the tumor microenvironment. Six LNP candidates with different compositions (100 mol% phosphatidylcholine (PC), 90 mol% PC and 10 mol% PEG_2000_-DSPE (10PEG), 90 mol% PC and 10 mol% phosphatidylserine (10PS), 80 mol% PC and 20 mol% PS (20PS), 90 mol% PC and 10 mol% DOTAP (10DOTAP), 80 mol% PC and 20 mol% DOTAP (20DOTAP)) were prepared through the film casting method to figure out the most preferable LNP for MSC-guided intratumoral drug delivery (Fig. 1d). Briefly, the solution of each lipid composition in 1 mL of chloroform was evaporated to cast a thin lipid film, and the film was hydrated with 1 mL of 3’DW and sonicated to obtain LNPs. All LNP candidates contained 0.5% Flamma 648-PE for their in vitro and in vivo fluorescent imaging analysis. First, the sizes of formulated LNP candidates in PBS were analyzed by DLS, exhibiting no significant difference depending on their compositions (Fig. 1e). Their sizes similarly ranged between 100 to 120 nm, and the size distributions were also monodisperse. In the zeta potential assay of LNPs, neutral PC showed a slightly positive value of + 7.67 ± 0.52 mV and 10PEG was also negatively charged to -24.43 ± 0.68 mV since PEG_2000_-DSPE has a phosphate group in its molecular structure. PS-containing LNPs (10PS and 20PS) showed negative surface charges (-40.23 ± 0.50 and -45.70 ± 0.79 mV, respectively) due to the negatively charged head group of PS, whereas 10DOTAP and 20DOTAP were highly positive (+ 39.97 ± 1.76 and + 46.50 ± 1.08 mV, respectively) due to the positively charged quaternary ammonium group in DOTAP [39–41]. All LNPs were furtherly observed with cryoTEM and determined to have similar sizes with spherical structures and uniform single-bilayer shells (Fig. 1f). When dispersed in PBS at a concentration of 1 mg/mL for up to 6 days, all LNPs stably secured their sizes without significant dissociation or aggregation (Fig. 1g).

### In vitro cellular uptake and cytotoxicity of LNPs

**Fig. 2:**
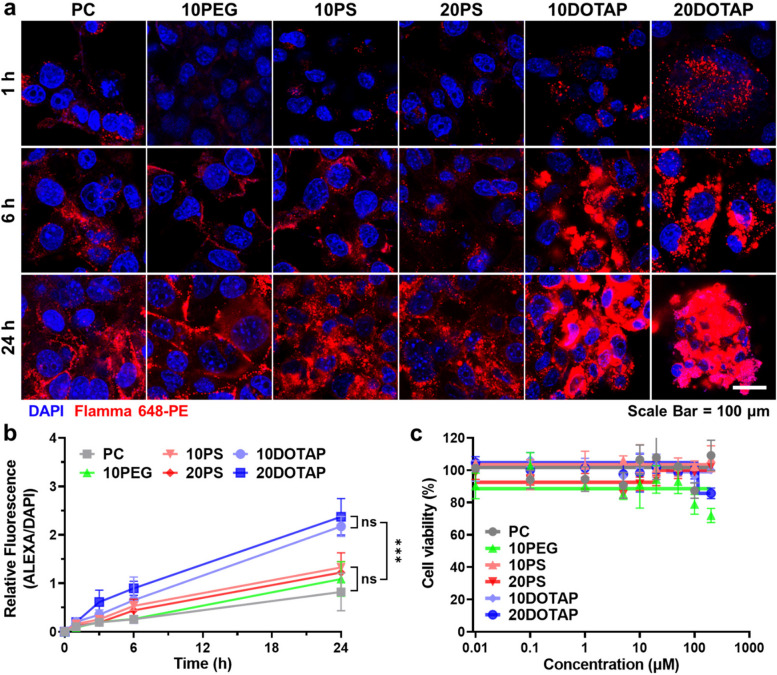
In vitro cellular uptake profile of LNP candidates (**a**) 4T1 cells were treated with six LNPs (0.1 mg/mL) and their endocytosis was observed via CLSM for 24 h, showing increasing intracellular fluorescent signals over time. **b** The fluorescent intensities of endocytosed LNPs were quantified and plotted as a function of time (*n* = 3). **c** 4T1 cells were treated with six LNPs at various concentrations from 0.01 to 200 μM and conducted with CCK-8 assay, expressing no significant toxicity. ns = not significant, ***p < 0.001

The formulated six different LNPs were subsequently applied to cancer cells to examine their cellular uptake profiles and cytotoxicity. To evaluate the cellular uptake of LNPs, 5 × 10^4^ 4T1 mouse breast cancer cells were seeded and treated with 0.1 mg/mL of each LNP and monitored using the CLSM for 24 h (Fig. 2a). The fluorescent signals by LNPs (red color) in the cytosol were gradually increased over time and reached their maxima at 24 h. The nano-sized PC, 10PEG, 10PS, and 20PS were moderately taken up by cancer cells regardless of their surface properties, showing a modest fluorescence level without a statistically significant difference (Fig. 2b). Their cellular uptake profile showed a linearly proportional correspondence to their treatment time. Notably, fluorescent signals of PC, 10PEG, 10PS, and 20PS were exclusively distributed in the cytoplasm rather than nuclei since LNPs are not permeable to the nuclear membrane [42]. Meanwhile, the fluorescent intensities of 10DOTAP and 20DOTAP were highest among the LNPs, showing 1.64 ~ 2.89-fold stronger signals than the other four LNPs. The positive surface charge of 10DOTAP and 20DOTAP was supposed to facilitate their interaction with negatively charged cell membranes and lead to their vigorous cellular uptake [43, 44]. However, their in vitro fluorescent signals were clumped and largely overlapped with the DAPI signal, which was not detected in other LNPs. It appeared that the low serum stability of 10DOTAP and 20DOTAP caused their partial agglomeration into submicron clusters inside the culture media and the clusters were attached to the cell membrane due to their strong charges (Fig. S2). Afterward, 4T1 cancer cells were treated again with LNPs at various concentrations ranging from 0.01 to 200 μM for their cytotoxicity assessment (Fig. 2c). All LNPs constantly showed negligible toxicities up to 100 μM, demonstrating their high biocompatibility as drug carriers. The endocytosis of LNPs mainly relied on their surface charges, which could not fully represent their accumulation and distribution inside tumor tissues since tumor microenvironments were not adequately implemented through the in vitro cellular assay. Although 10DOTAP and 20DOTAP expressed the most active interaction with cancer cells and intense endocytosis, their surface properties might not be appropriate for the intratumoral distribution. Therefore, actual delivery behaviors of LNPs with MSC guidance were investigated in the following in vivo test using tumor-bearing mice.

### MSC-guided tumoral accumulation of LNPs in tumor-bearing mice

**Fig. 3:**
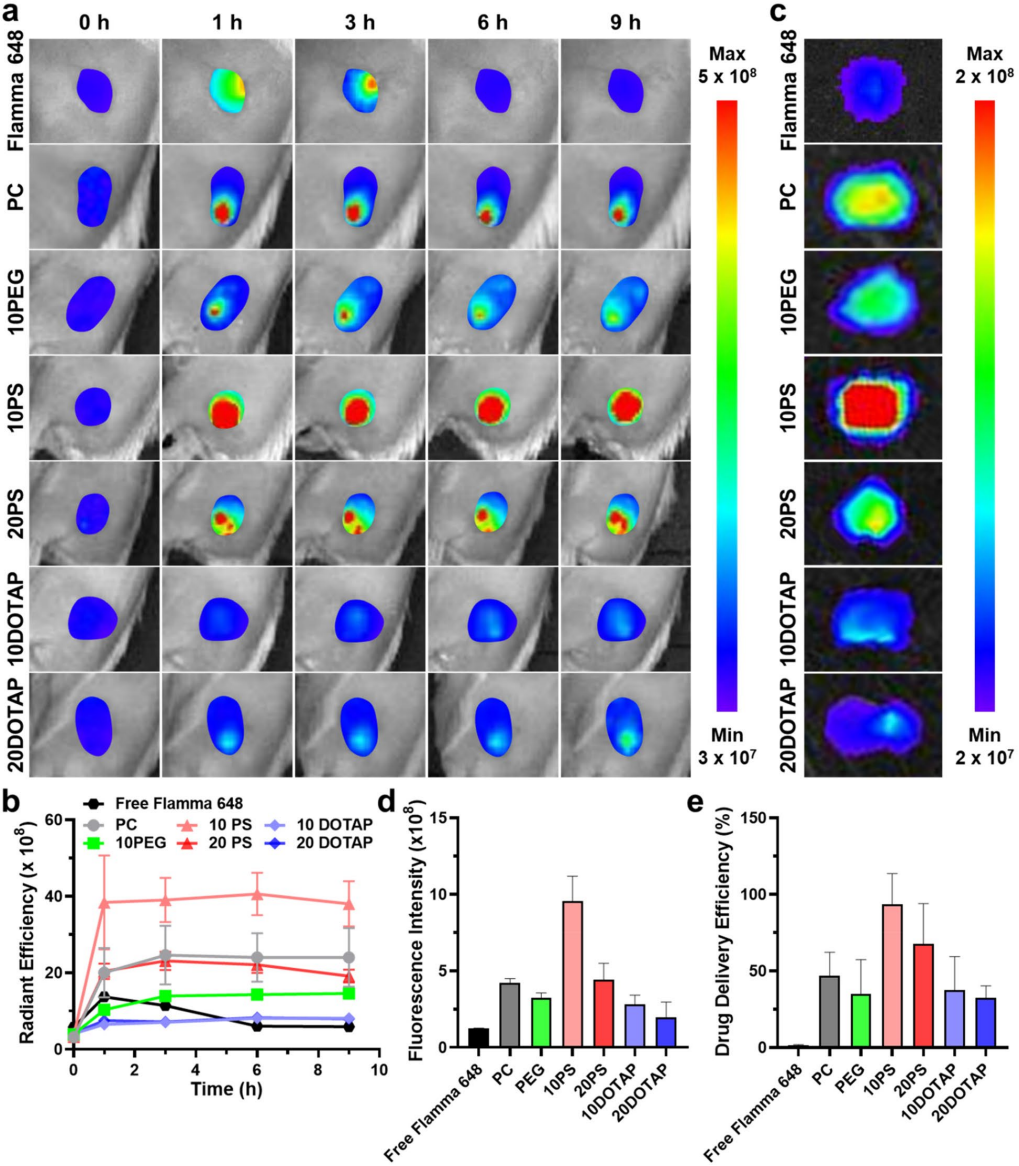
In vivo intratumoral accumulation analysis of LNP candidates administered via MSC guidance (**a**) The IVIS of 4T1 tumor-bearing mice treated with six LNPs (30 mg/mL, 1 μL) or free Flamma 648 dye (0.15 mg/mL, 1 μL) via MSC guidance showed the time-dependent intratumoral accumulation behaviors of LNPs and Flamma 648 dye. **b** The in vivo intratumoral fluorescent intensity was measured over the administration time, showing the highest accumulation of 10PS. **c** The ex vivo fluorescence images of excised tumor tissues 9 h after the MSC-guided LNP treatment were obtained and (**d**) their fluorescent intensities were quantified. **e** The intratumoral delivery efficiencies of six LNPs and Flamma 648 dye were calculated by the ratios of tumor-remaining LNP or dye concentrations over those released from the MSC

It was hypothesized that the surface properties of LNPs affect their accumulation and distribution inside the tumor tissue as they were administered using the MSC. To discover the most appropriate LNP for the maximal and uniform delivery of drugs throughout the tumor tissue, tumor-bearing mice were prepared and LNP-loaded MSCs were applied directly to tumor tissues to monitor the release and distribution of LNPs. The MSC-guided intratumoral administration of free Flamma 648 dyes was also conducted to compare the intratumoral accumulation behavior of LNPs with that of small molecules and determine the effect of LNP encapsulation on the intratumoral administration. Tumor-bearing mouse models were constructed by subcutaneously inoculating 1 × 10^6^ 4T1 cells in the left thighs of BALB/c mice. After the tumor volumes reached ~ 200 mm^3^, MSCs containing 1 μL of each LNP (30 mg/mL) or Flamma 648 solution (0.15 mg/mL) were directly implanted into the center of tumor tissues, and the release and localization of LNPs or free dye were non-invasively observed via fluorescence imaging device for 9 h. The implanted MSCs were protected by plastic caps to prevent any contamination or damage to the MSC (Fig. S3). When comparing the fluorescent signals of LNPs inside MSCs before and after their administration, the percentages of LNPs released out from MSCs were about 50 ~ 60%, similar to one another regardless of LNP compositions (Fig. S4). The LNPs were gradually released from MSC and diffused into tumor tissues over 9 h, which was detectable via in vivo fluorescence imaging of tumors (Fig. 3a). The fluorescences of administered LNPs from MSC were observed exclusively inside the tumor tissues, and the signals were maintained during the 9 h of infusion procedure. However, the intratumoral fluorescence of free Flamma 648 dye reached its highest at 3 h post-administration, and the intratumorally remaining dye was not observed after 6 h. The MSC-guided intratumorally administered free Flamma 648 dye was considered to rapidly diffuse through the tumor interstitium and washed out from tumor tissues due to the excessive diffusivity of small molecules [45]. LNPs were more advantageous for MSC-guided intratumoral administration than free dye due to their moderate intratumoral diffusivity and prolonged retention. It was notable that the intratumoral accumulation of LNPs was closely related to their surface properties. Neutral PC and negative 10PS and 20PS rapidly accumulated at targeted tumor tissues within 1 h post-administration, wherein the bright red fluorescent signals of LNPs were clearly observed in tumor tissues. Furthermore, most of their fluorescent signals were maintained up to 9 h post-administration. In particular, 10PS was freely diffused into the tumor and expressed the brightest fluorescence compared to other LNPs. On the contrary, the fluorescence of 10PEG gradually spread to the entire tumor tissue over time, but its intratumoral accumulation at 9 h was not high because of its extratumoral leakage. Moreover, positively charged 10DOTAP and 20DOTAP did not show perceptible fluorescent signals at tumor tissues for 9 h, indicating the lowest tumor targeting ability of positively charged LNPs. When quantifying the intratumoral fluorescent intensity over time, the tumor infusion rates of LNPs were most rapid in the first 1 h and saturated at 6 h (Fig. 3b). Noticeably, the fluorescent intensity of 10PS was the highest throughout the infusion time, which was 1.58 ~ 4.80-fold higher compared to those of other LNPs at 9 h post-administration. However, the positively charged 10DOTAP and 20DOTAP showed low fluorescent intensity over 9 h of MSC-guided administration, and that of free Flamma 648 dye was the minimum due to its fast diffuse-out.

After 9 h post-administration, tumor tissues were excised for a more precise analysis of intratumoral LNP and free Flamma 648 localization. The strongest intratumoral fluorescent signal of 10PS was clearly observed in tumor tissues, while free Flamma 648 rarely remained inside the tumor tissues (Fig. 3c). The fluorescent intensity of 10PS was 2.2 ~ 4.9-fold higher than those of other LNPs, whereas 10DOTAP and 20DOTAP showed the lowest fluorescent signals among the LNPs (Fig. 3d). To determine the actual delivery efficiency of LNPs from MSC, each tumor was homogenized and the amount of each LNP in the entire tumor tissues was measured using the high-performance liquid chromatography (HPLC). As expected, 10PS exhibited a high delivery efficiency of 93%, which was calculated by the ratios of LNP concentrations inside tumor tissues to those released out from MSCs (Fig. 3e). However, other LNPs showed decreased delivery efficiencies from 32 to 68% in the HPLC analysis, and the delivery efficiency of free Flamma 648 was merely less than 2%. Based on these data, the negatively charged 10PS was determined as the most desirable LNP for the MSC-guided intratumoral administration, while positively charged or PEGylated LNPs can hinder their efficient localization inside tumors.

**Fig. 4:**
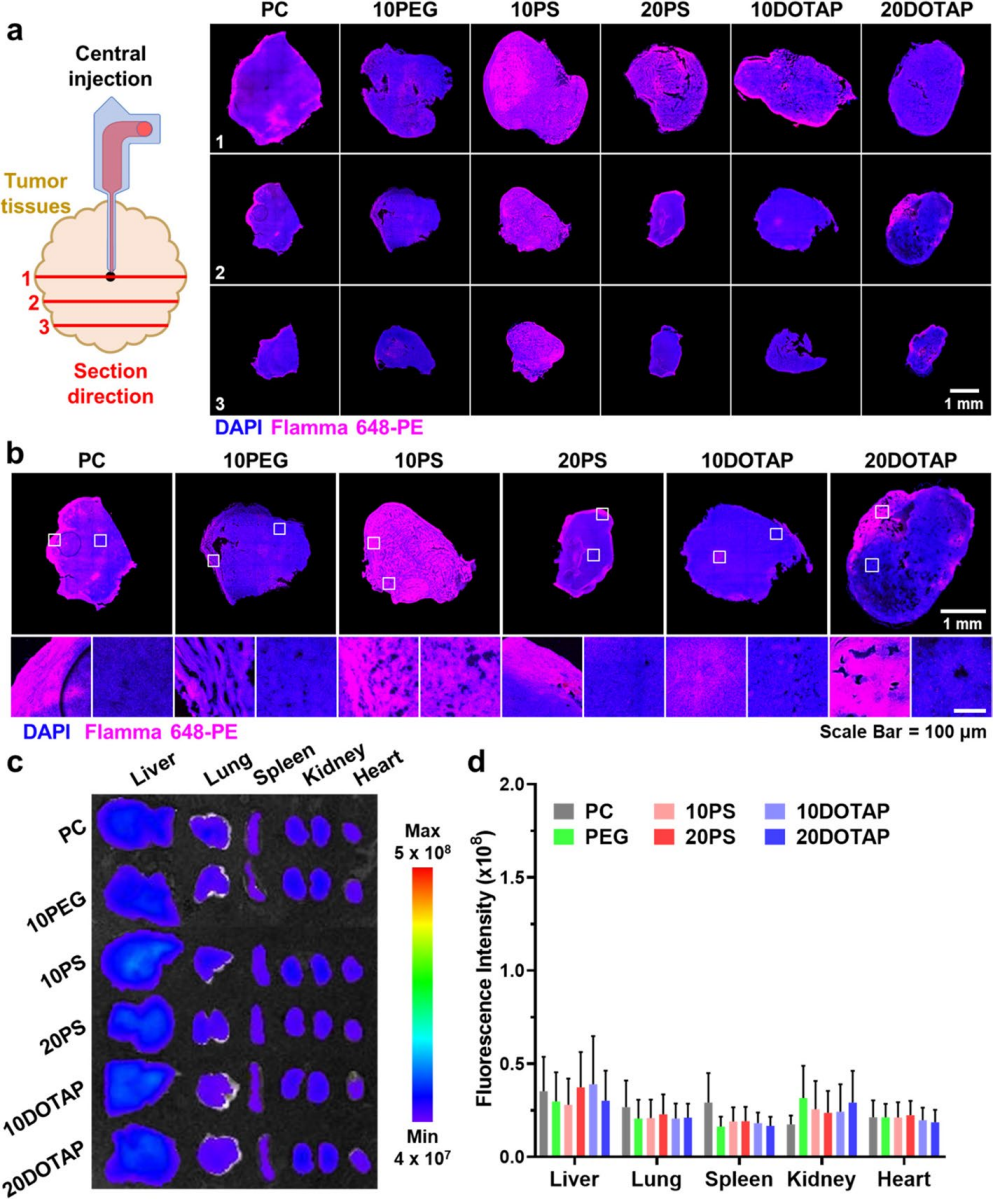
Ex vivo intratumoral distribution assay of MSC-guided administered LNP candidates (**a**) The CLSM images of tumor sections from three different regions were acquired, determining that the 10PS-treated tumor tissue expressed the highest fluorescent signals throughout all sections. **b** The brightest and darkest areas in the CLSM images of Sect. 2 were magnified to precisely compare the regularity of LNP distribution inside the tissues. 10PS was found to be most evenly distributed inside the tumor. **c** The extracted organs and tumors from all recipient mice were examined with IVIS and (**d**) their fluorescent intensities were quantified

The excised tumors were subsequently chopped into three tissues with a 1 mm-thickness interval to observe the intratumoral distribution of LNPs. Each section was stained with DAPI (nuclei staining, blue color) and analyzed with CLSM, showing the specific localization of Flamma 648-PE-labeled LNP fluorescent signals (red color) (Fig. 4a). In the confocal whole-section images of Sect. 1, the central region of MSC administration, the tumor tissues treated with PS, 10PS, 20PS, 10DOTAP, and 20DOTAP showed the concentrated LNP accumulation in Sect. 1, whereas 10PEG-treated tumor tissue showed the minimum fluorescent signal, indicating the lowest localization in tumor tissues. Notably, only 10PS showed bright and uniform fluorescence distribution throughout Sect. 2 and Sect. 3, indicating that negatively charged LNPs with 10 mol% PS might freely diffuse from the center of MSC administration to whole tumor tissues and then be robustly taken up by cancer cells before eluding out at targeted tumor tissues. In contrast, LNP-administered tumors except 10PS showed relatively dim fluorescence in Sect. 2, marginally apart from the administration site, and the intensity was further decreased at the distal Sect. 3. The diffusivity of PC, 10DOTAP, and 20DOTAP was considered not adequate for their intratumoral distribution. Notably, all three sections of 10PEG-treated tumors were the lowest in fluorescence level since 10PEGs were diffused out from the tumor tissue rather than remaining inside it due to the PEGylation effect. When magnifying the confocal images of Sect. 2, tumor tissues treated with other LNPs than 10PS had mottled fluorescence patterns due to the skewed distribution of LNPs (Fig. 4b). Considerable differences in fluorescent signals between LNP-concentrated and sparse regions were observed, indicating their inhomogeneous localization. The partial accumulation of 10DOTAP and 20DOTAP was clearly noticeable (white square), being attributed to their aggregation in the physiological condition and thereby abnormal diffusion in whole tumor tissues. In the case of 10PS-treated tumor tissue, however, the fluorescent signal was uniformly distributed and the regional fluorescent intensity gap between the highest and the lowest was ignorable. As a result, 10PS was finally chosen as the most adequate LNPs for MSC-guided drug delivery since it could be efficiently taken up by cancer cells, highly accumulated inside tumors, and uniformly diffuse throughout whole tumor tissues.

Fluorescent signals from normal organs were additionally analyzed ex vivo to confirm any undesirable delivery of LNPs in normal tissues (Fig. 4c). The fluorescent signals of all LNPs were barely detectable from all organs regardless of the MSC-guided administered LNP types, indicating that the non-specific accumulation of LNPs in normal tissues was ignorable. The quantified fluorescent intensities of the organs also exhibited no statistical difference from one another (Fig. 4d). The utilization of MSC for the intratumoral LNP delivery allowed LNPs to be slowly infused into tumor tissues via their physiological diffusion mechanism, providing them sufficient time for 9 h to spread throughout the tumor tissues. Moreover, LNPs could be administered precisely into the center of tumors through the microchannel of MSC, which promoted their homogenous diffusion to the tumor periphery without any inappropriate delivery to normal tissues. In addition to the infusion control by MSC guidance, the surface chemistry of LNPs further affected their intratumoral distribution, wherein highly cationic LNPs were unevenly dispersed and PEGylated LNPs tend to rapidly elude out from tumors. These data indicate that MSC-guided intratumoral administration of LNPs can solve the serious problems of intravenously administered LNPs such as interactions with blood components and entrapment to the reticuloendothelial system (RES) that greatly disturb their passive tumor targeting at targeted tumor tissues.

### Characterization and in vitro cellular assay of ApoLNPs

**Fig. 5:**
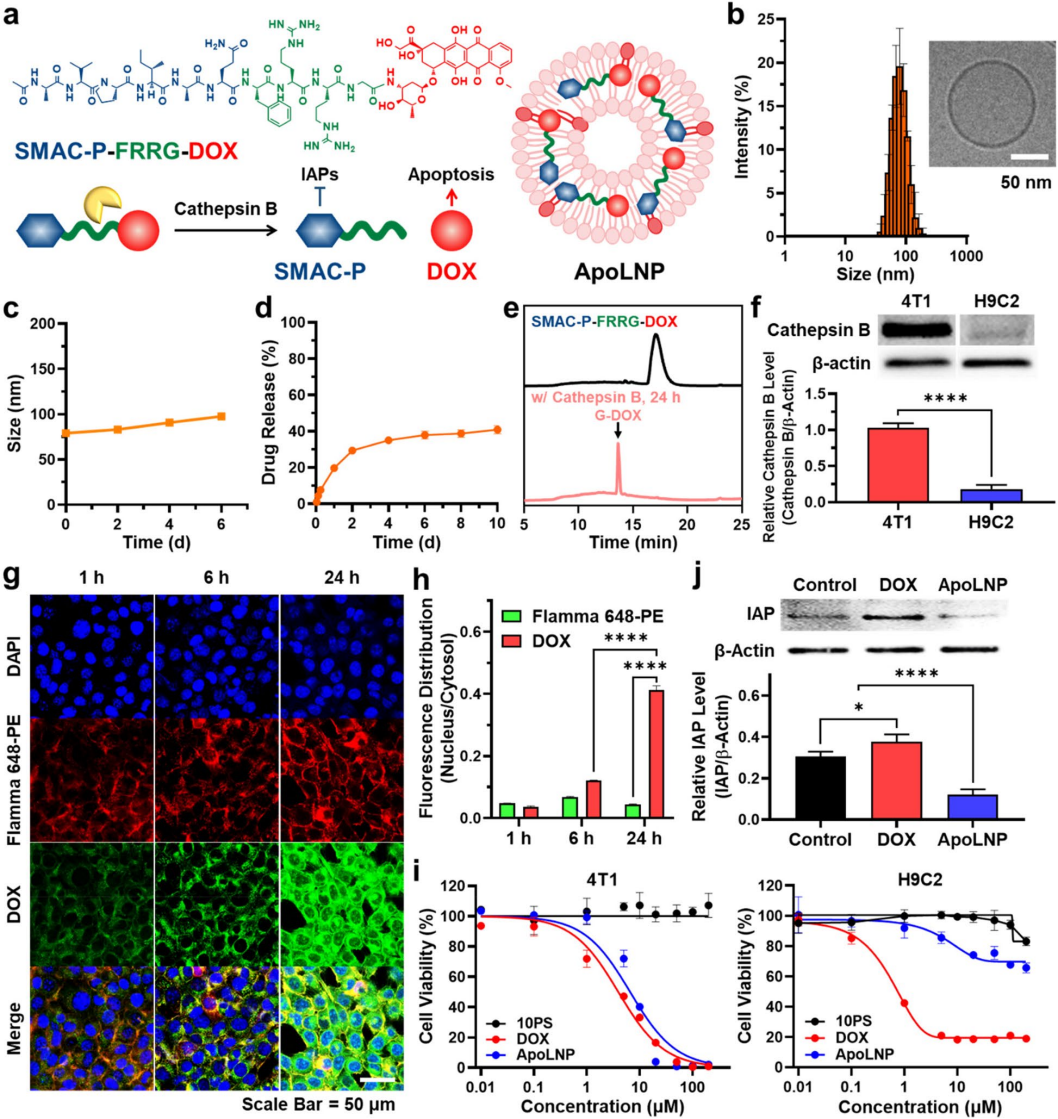
In vitro characterization of ApoLNP (**a**) ApoLNP was prepared by encapsulating the SMAC-P-FRRG-DOX prodrug inside the 10PS. **b** The formed ApoLNP (1 mg/mL) was assessed via DLS and cryoTEM, confirming a similar size and morphology to that before SMAC-P-FRRG-DOX loading. **c** The dispersion stability of ApoLNP in PBS buffer (1 mg/mL) was observed for up to 6 days using DLS. **d** The ApoLNP (15 mg/mL) was placed into a dialysis bag (MWCO = 12–14 kDa) and dialyzed with 10 mL PBS for 10 days to examine the release behavior of SMAC-P-FRRG-DOX. **e** The HPLC analysis of SMAC-P-FRRG-DOX (0.1 mM) before and after the incubation with cathepsin B (10 μg/mL) displayed its cathepsin B-specific cleavage into SMAC-P-FRR and G-DOX (**f**) The excessive cathepsin B expression level of 4T1 cancer cells compared to that of H9C2 normal cells was analyzed via western blot assay (*n* = 3). **g** 4T1 cells were treated with ApoLNP (5 μM, based on SMAC-P-FRRG-DOX content) and their CLSM analysis was carried out at 1, 6, and 24 h after the treatment to determine its endocytosis and intracellular distribution. **h** The ratios of LNPs (Flamma 648-PE, red color) and DOXs (green color) localized inside the cytosol and nuclei were compared to confirm the increased intranuclear distribution of DOXs over time (*n* = 3). **i** 4T1 cancer and H9C2 normal cells were exposed to various concentrations of ApoLNPs, DOXs, and 10PSs from 0.01 to 200 μM and their CCK-8 assay was conducted to prove the selective toxicity of ApoLNP to cathepsin B-overexpressing cancer cells. **j** The reduced IAP expression level of ApoLNP-treated 4T1 cells over DOX-treated ones was determined via the western blot (*n* = 3). * *p* < 0.05, **** *p* < 0.0001

For the MSC-mediated cancer therapy, cathepsin B-cleavable and pro-apoptotic prodrugs (SMAC-P-FRRG-DOXs) were encapsulated into 10PS, which had been determined as the optimal LNP for intratumoral administration, resulting in ApoLNPs (Fig. 5a). The SMAC-P-FRRG-DOX prodrug is a conjugate of second mitochondria-derived activator of caspases mimetic peptides (SMAC-P; *AVPIAQ*) and DOX with a cathepsin B-cleavable peptide linker (*FRRG*). The SMAC-P-FRRG-DOX is subsequently cleaved to SMAC-P and DOX in cathepsin B-overexpressing cancer cells. It has been already reported that SMAC-P-FRRG-DOX showed a significant antitumor efficacy owing to the synergistic activity of the inhibitor of apoptosis proteins (IAPs) antagonism with chemotherapy in drug-resistant breast tumor models [46]. The SMAC-P-FRRG-DOX was successfully synthesized via the simple esterification coupling of SMAC-P-FRRG and DOX, which was confirmed through HPLC and LC–MS analysis (m/z = 841.7, [M]/2 + 1H^+^ and 1683.2, [M] + 2H^+^) (Fig. S5). Subsequently, 3 mg of SMAC-P-FRRG-DOX was loaded into 30 mg/mL 10PS through the film casting method, and the loading efficiency of SMAC-P-FRRG-DOX was 98% [47]. The freshly prepared ApoLNPs were dispersed in PBS at 1 mg/mL and analyzed via DLS, determined to have a mean diameter of 78.95 ± 0.96 nm (Fig. 5b). The cryoTEM image of ApoLNPs exhibited its spherical and single-bilayered structure, similar to that of 10PS before the SMAC-P-FRRG-DOX encapsulation. In addition, the size and polydispersity of ApoLNPs in PBS were stably maintained for up to 6 days (Fig. 5c). The release profile of SMAC-P-FRRG-DOX from ApoLNPs was monitored for 10 days and 40% of SMAC-P-FRRG-DOX slowly released for 4 days (Fig. 5d). The cathepsin B-responsive degradation of SMAC-P-FRRG-DOX was further confirmed in bench condition (Fig. 5e). When 0.1 mM SMAC-P-FRRG-DOX was incubated with 10 μg/mL cathepsin B for 24 h and analyzed via HPLC, the peak corresponding to SMAC-P-FRRG-DOX completely disappeared and the new peak of G-DOX newly emerged. The cleaved G-DOX was analyzed via LC–MS again, confirming the consistency of m/z to its theoretical value (m/z = 623.3, [M] + Na^+^) (Fig. S6). The G-DOX cleaved from SMAC-P-FRRG-DOX was successfully metabolized into free DOX by intracellular proteases in lysosomes [48, 49]. Next, 4T1 cancer cells were treated with ApoLNPs to investigate their cellular uptake behavior and cytotoxicity. Firstly, 5 × 10^4^ 4T1 cells were seeded and cultured with ApoLNPs containing 5 μM of SMAC-P-FRRG-DOX and observed via CLSM at 1, 6, and 24 h. As expected, 4T1 cancer cells expressed 5.6-fold higher cathepsin B compared to normal cells of rat cardiomyocytes (H9C2), indicating that the prodrug of SMAC-P-FRRG-DOX in ApoLNPs can be specifically cleaved via cancer cell-overexpressed cathepsin B (Fig. 5f) [46]. The fluorescent signals of DAPI (nuclei, blue color), Flamma 648-PE (10PS LNPs, red color), and DOX (SMAC-P-FRRG-DOX or free DOX, green color) were separately obtained to evaluate time-dependent cellular uptake and intracellular localization of ApoLNPs (Fig. 5g). The fluorescent signal of ApoLNPs in cytosols continuously increased over time and was maximized at 24 h, showing the similar cellular uptake behavior to that of 10PS. Notably, the fluorescence of DOX mainly existed in cytosols rather than nuclei at 1 and 6 h, but some of it could be also detected inside nuclei at 24 h. The ratio of DOXs inside the nuclei and cytosol at 24 h was 0.41 ± 0.01, 3.4-fold higher than that at 6 h (Fig. 5h). It was considered that SMAC-P-FRRG-DOXs were slowly released from ApoLNPs and cleaved by cathepsin B to SMAC-P and free DOX [46]. On the contrary, the fluorescent signal of 10PS (green color) was rarely found in nuclei since LNPs could not penetrate the nuclear membrane. The ratio of intranuclear and cytosolic 10PS during the cellular uptake assay did not exceed 0.07 and it was 9.2-fold lower than that of DOX at 24 h. The cell-internalized ApoLNPs induced cancer-specific and pro-apoptotic cell death due to cancer cell-overexpressed cathepsin B cleavage mechanism of SMAC-P-FRRG-DOX (Fig. 5i). The IC_50_ value of ApoLNPs against 4T1 cells was 6.6 μM, with a slight difference from that of free DOX (3.9 μM) due to the delayed drug release from ApoLNPs. In contrast, ApoLNPs showed greatly reduced toxicity to H9C2 rat normal heart cells with 230.7 μM IC_50_ value, whereas free DOX was substantially toxic to normal cells (IC_50_ = 1.1 μM). Moreover, ApoLNPs efficiently suppressed the DOX-induced drug resistance through the IAPs antagonism by SMAC-P released from ApoLNPs. In the western blot assay, 4T1 cells treated only with DOX expressed a 1.2-fold excessive level of inhibitors of IAPs compared to the saline-treated ones, which regulates programmed cell death of cancer cells (Fig. 5j) [50]. Meanwhile, ApoLNP-treated cells had a relatively low IAPs expression level since the co-delivered SMAC-P directly blocked IAPs and led them to be degraded [51]. The level of IAPs of ApoLNP-treated cancer cells was 3.1-fold lower than that of DOX-treated 4T1 cells. Therefore, ApoLNPs demonstrated their highly specific pro-apoptotic cell death induction in cathepsin B-overexpressing cancer cells.

### In vivo tumor accumulation of ApoLNPs in 4T1 tumor-bearing mice

**Fig. 6:**
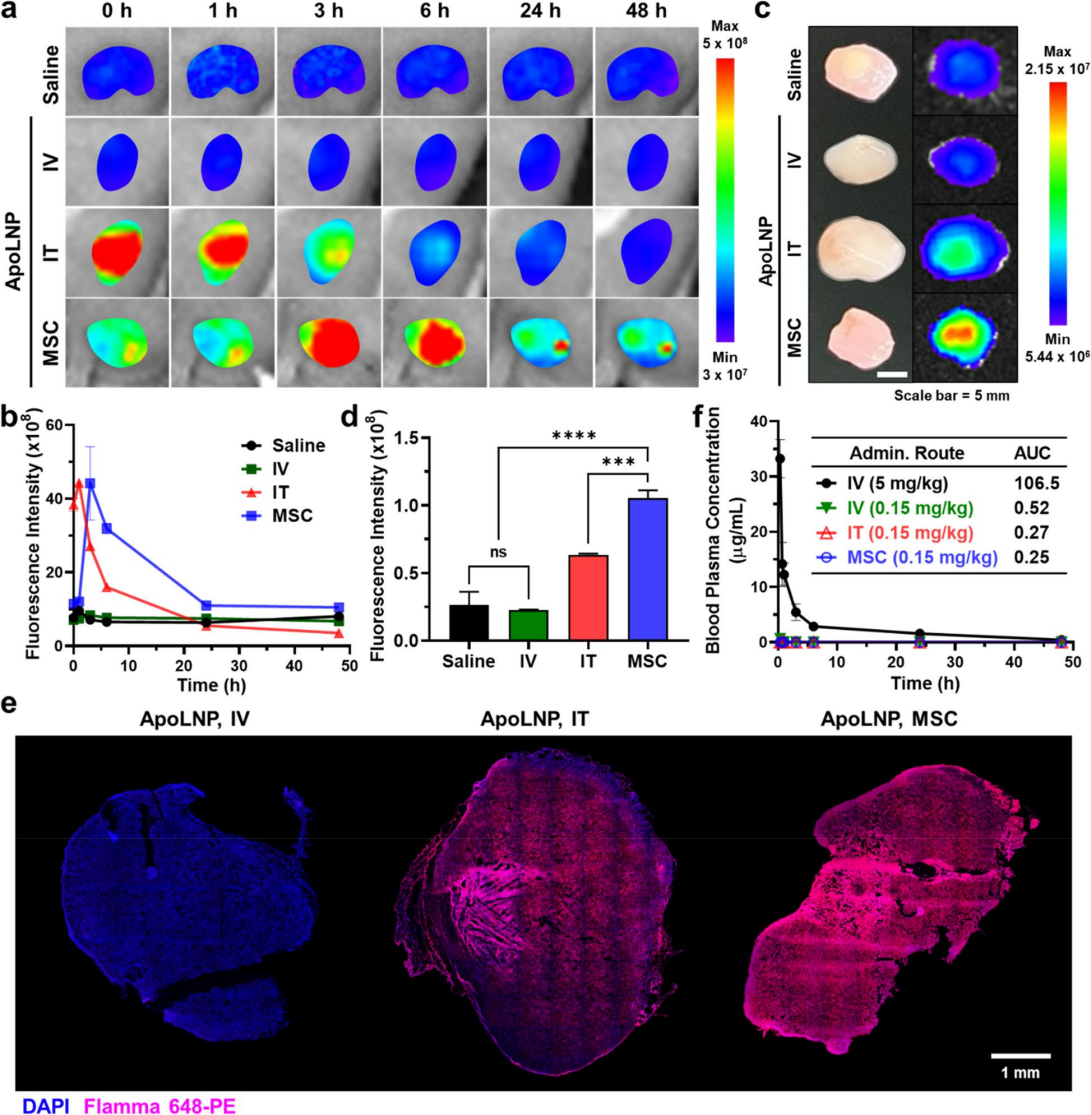
In vivo investigation for the intratumoral accumulation of ApoLNP with different administration routes (**a**) 4T1 tumor-bearing mice were administered with ApoLNPs (0.15 or 3 mg/kg, based on DOX content) via IV, IT, and MSC-guided routes and their IVIS images were collected for 48 h (fluorescence signals of Flamma 648-PE in 10PS). **b** The in vivo tumor fluorescent intensities were calculated and described as a function of time. **c** After 48 h of intravenous, intratumoral, and MSC-guided ApoLNP treatment, tumor tissues were extracted to take their ex vivo IVIS images, and (**d**) their fluorescent intensities were measured (*n* = 3). **e** The distribution of ApoLNPs inside IV, IT, and MSC-guided administered tumors was assessed using the CLSM. **f** The pharmacokinetics of ApoLNPs administered via different routes were analyzed by quantifying the changes in blood plasma concentrations of ApoLNPs over 48 h through the HPLC. ns = not significant, *** *p* < 0.001, **** *p* < 0.0001

The tumor accumulation of ApoLNPs depending on their administration routes was investigated using 4T1 tumor-bearing mice. 1 × 10^6^ 4T1 cells were subcutaneously administered into BALB/c mouse models, then as tumor tissues grew to ~ 200 mm^3^, ApoLNPs (0.15 mg/kg, based on DOX content) were administered to the mouse models through different routes (intravenous (IV), intratumoral (IT), or MSC-guided). The different accumulations of ApoLNPs inside tumor tissues were monitored in vivo for 48 h via an IVIS imaging system, visualizing the fluorescence signals by LNPs (Flamma 648-PE) (Fig. 6a). In the case of IV administration, ApoLNP was hardly delivered to tumor tissues thereby the intratumoral fluorescence was not different from that with saline administration, indicating the poor delivery efficiency of the systemic administration. In the meantime, the direct IT administration exhibited significantly higher tumor accumulation of ApoLNP than its IV administration, but a large proportion of administered ApoLNP rapidly diffused out within 6 h, and no fluorescent signal was detected inside the tumor tissue at 24 h after administration. In particular, the MSC-guided administration for 6 h successfully facilitated the enhanced and long-lasting tumor accumulation of ApoLNP. The quantified data of time-dependent changes in tumor fluorescent intensity showed that the area under the curve (AUC) of MSC-guided administration was 584.8 ± 22.6, which was 5.1- and 2.3-fold larger than those of IV and IT administration, respectively (Fig. 6b). Although the peak fluorescent intensity of the MSC-guided administration was similar to that of the IT administration, it allowed the ApoLNPs to remain in the tumor more persistently. The immediate IT administration of ApoLNPs caused them to be excessively localized inside the tumor tissues at once, resulting in their leakage through the blood and lymphatic vessels before being uniformly dispersed and endocytosed. In contrast, the MSC-guided administered ApoLNPs could sufficiently diffuse all around the tumor tissues and be taken up by cancer cells during 6 h of infusion time, thereby achieving a higher and persistent tumor targeting efficiency.

The ex vivo images of tumors with different administration routes after 48 h also proved the effectiveness of MSC-guided administration (Fig. 6c). The fluorescent signal in the tumor with low-dose IV administration was the lowest, and the signal gradually became brighter in the order of IV, IT, and MSC-guided administration. The fluorescence of the tumor tissue with MSC-guided administration was 4.6- and 1.7-fold brighter than those of IV and IT administration, respectively. This was deduced that a considerable amount of intratumorally administered ApoLNPs leaked out from the tumor whereas those administered by MSC guidance still remained inside the tumor tissue (Fig. 6d). The intratumoral accumulation of ApoLNPs was further confirmed via the CLSM analysis of tumor tissues (Fig. 6e and S7). After 48 h post-administration, the tumor tissue with MSC-guided ApoLNP administration expressed the strongest fluorescence which was 2.2-fold higher than the IT-treated one, whereas the fluorescence was not detectable in the IV-treated tumor (Fig. S8). In addition, ApoLNPs were most uniformly distributed inside the whole tumor tissue when they were administered using the MSC. The MSC-guided administration of ApoLNPs showed the promoted tumor targeting efficiency at targeted tumor tissues compared to IV and IT administration.

To investigate the effect of LNPs on MSC-guided intratumoral administration, free DOXs, SMAC-P-FRRG-DOXs, and ApoLNPs were intratumorally administered to 4T1 tumor-bearing mice at their doses of 0.15 mg/kg (based on DOX content) through the MSC guidance and their intratumoral accumulation was observed for 48 h via the IVIS imaging. In the case of the ApoLNP administration, the fluorescence signals by DOXs could be detected for a prolonged period of up to 48 h, demonstrating the enhanced intratumoral retention of ApoLNPs due to the moderate diffusivity of 10PSs (Fig. S9a). In contrast, MSC-guided intratumorally administered free DOXs smoothly were released into tumor tissues for 3 h but they were rapidly diffused out from tumor tissues, thereby no fluorescence signal by DOX was visible after 6 h of administration. SMAC-P-FRRG-DOXs could not remain inside the tumor tissues longer than 6 h either, indicating that the LNP encapsulation is essential for the persistent intratumoral localization of drugs in their MSC-guided administration. The intratumoral fluorescence intensity of ApoLNPs at 48 h post-administration was 1.9- and 3.2-fold higher than those of SMAC-P-FRRG-DOXs and free DOXs, respectively (Fig. S9b). The intratumorally remaining concentration of SMAC-P-FRRG-DOXs was considered slightly higher than that of free DOXs due to their higher molecular weight and lower diffusivity, but not sufficient compared to ApoLNPs. The utilization of LNPs in the MSC-guided intratumoral administration was proven to improve the long-term tumor-specific localization of drugs, which would directly affect their therapeutic efficacy and systemic toxicity.

After determining the intratumoral accumulation of ApoLNPs depending on their administration routes, their pharmacokinetic behaviors and distribution in normal organs were analyzed over time. When tracking their plasma concentration for 48 h, the concentrations of ApoLNPs with IV, IT, and MSC-guided administration at 0.15 mg/kg doses were significantly low in blood samples of recipient mice since their doses were about 20-fold lower than the typical intravenous administration (Fig. 6f). The AUC of IV, IT, and MSC-guided administered ApoLNPs at 0.15 mg/kg doses were 0.52, 0.27, and 0.25, respectively, 204.8 ~ 426.0-fold lower than that of IV-administered ones at a 5 mg/kg dose. Further, the AUC of MSC-guided administered ApoLNPs was 2.1-fold lower than that of IV-administered ones at the same doses, confirming the prolonged tumor retention and diminished extratumoral leakage of ApoLNPs via their MSC-guided administration. In the ex vivo fluorescence images of normal organs, the fluorescence signals by MSC-guided administered ApoLNPs were rarely detected, thereby their accumulation to non-target tissues was considered ignorable (Fig. S10). Those results in pharmacokinetic profiles and biodistribution assessment of ApoLNPs indicated that the MSC-guided administration can effectively prohibit the undesirable distribution of ApoLNPs in normal tissues by delivering them tumor-exclusively and reducing their required minimal doses.

### Therapeutic efficacy of MSC-guided administered ApoLNPs against tumor-bearing mice

**Fig. 7:**
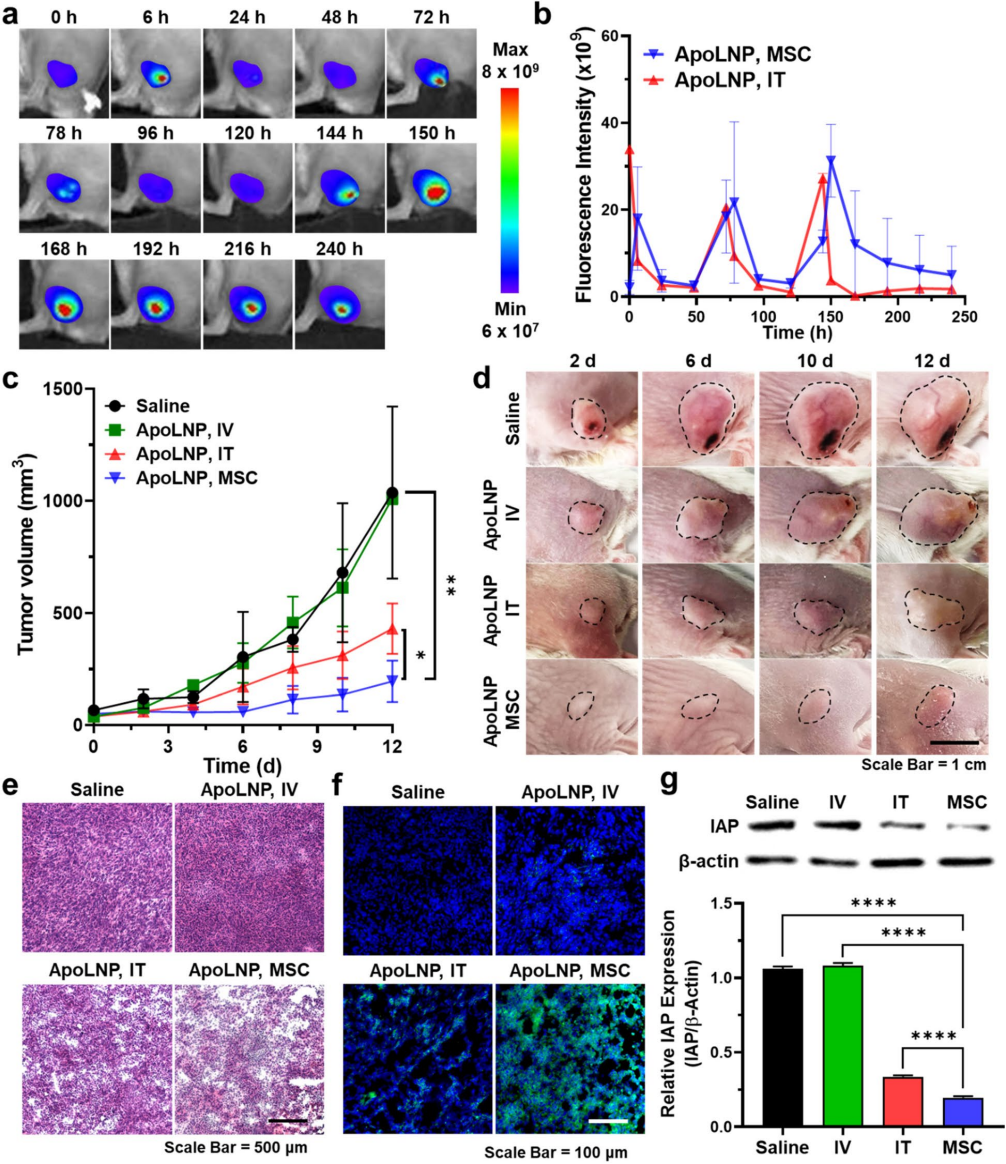
In vivo therapeutic efficacy comparison of ApoLNP with different administration routes (**a**) During the MSC-guided ApoLNP treatment (0.15 mg/kg, based on DOX content, once per 3 days), the IVIS images of recipient mice were acquired and (**b**) the intratumoral fluorescent intensities were calculated. **c** The tumor sizes were measured for 12 days to compare the difference in therapeutic effect according to the ApoLNP administration routes, and (**d**) their digital images were also obtained (black dashed line = tumor) (*n* = 3). **e** Upon completing the treatment procedure, tumor tissues were collected and their H&E histology was performed. **f** The tumor tissues were further stained with TUNEL to determine the degree of tumor apoptosis. **g** The tumoral IAP expression levels depending on the ApoLNP administration routes were evaluated via western blot (*n* = 3). * *p* < 0.05, ** *p* < 0.01, **** *p* < 0.0001

The therapeutic efficacy of ApoLNPs with MSC-guided administration was assessed via their repeated application to tumor-bearing mice and compared to that with the IT and IV administration. When 4T1 tumor tissues grew to ~ 100 mm^3^, ApoLNPs (0.15 mg/kg, based on DOX content) were administered to the mouse models once per three days (three times in total), wherein MSC-guided administration was carried out for 6 h. The repeated ApoLNP administration was continually observed through fluorescence imaging. As expected, ApoLNPs could be smoothly administered into tumor tissues three times through both IT and MSC-guided administration (Fig. 7a and S11). The fluorescent intensity of the MSC-administered tumor reached peak points 6 h after each administration and gradually increased in tumor tissues for 10 days, which was similar to the single administration profile (Fig. 7b). In contrast, the fluorescent intensity of the IT-administered tumor was decreased immediately after the administration, thereby the AUC of IT-administered tumors was 1.7-fold lower than the MSC-guided administered ones. Notably, MSC-guided administered tumors at 7–10 days exhibited 2.8 ~ 47.8-fold brighter fluorescent signals than those of IT administration, confirming the persistent intratumoral remaining of MSC-guided administered ApoLNPs.

To evaluate the therapeutic efficacy of ApoLNPs, the average tumor sizes were measured every two days and compared to those with saline treatment (black dashed line = tumor) (Fig. 7c and d). The MSC-guided ApoLNP administration successfully suppressed the tumor growth with 195.02 ± 75.65 mm^3^ of tumor sizes after 12 days. The tumors of the MSC-guided ApoLNP administration group were 4.7- and 2.2-fold smaller than those of saline-treated (1036.50 ± 313.44 mm^3^) and IT-administered (430.02 ± 91.80 mm^3^) groups, respectively, confirming the therapeutic effectiveness of MSC-guided administration. Noticeably, IV administration did not show any therapeutic efficacy, thereby the growth of IV-administered tumors was similar to that of the saline-treated ones. This was attributed to that the 0.15 mg/kg was too low compared to the conventional DOX dose for its intravenous administration, which is generally 1–3 mg/kg [46]. After the treatment, all experimental groups were sacrificed and their normal organs and tumor tissues were harvested for further analysis. The ex vivo fluorescence images of organs from MSC-guided ApoLNP administration groups showed not much difference in their fluorescent intensities compared to those from saline-treated groups even after three times repeated doses, indicating that no significant leakage and off-target accumulation of ApoLNPs were observed (Fig. S12). The IT-administered ApoLNPs were slightly detectable in the liver, which was not statistically significant. During the therapeutic procedure, all treatment groups did not show any notable weight loss (Fig. S13). This was attributed to the inactiveness of SMAC-P-FRRG-DOX against normal tissues that exhibit low cathepsin B expression levels. In addition, the histological images of normal organs from the three-times ApoLNP-treated groups did not exhibit significant damage regardless of the administration route (Fig. S14). The organs were preserved with their integrities similar to those from saline-treated groups, demonstrating the systemic safety of ApoLNPs.

The excised tumor tissues were further precisely analyzed by staining them with H&E, TUNEL, and IAPs antibodies. In the histological assay, MSC-guided ApoLNP-treated tumor tissues were determined to be seriously damaged due to the therapeutic effect of localized ApoLNPs (Fig. 7e). Moderate damages were also found in the tumors treated with IT administration of ApoLNPs, but not so intense as MSC-guided administration since the tumor accumulation of ApoLNP was not sufficient. The ApoLNP-induced tumor apoptosis was visible more obviously in the TUNEL images (Fig. 7f). The free DOX and SMAC-P released from ApoLNPs were thought to synergistically act in tumor tissues, leading to their severe apoptosis. Moreover, the IAPs expression was remarkably suppressed in the tissues with MSC-guided ApoLNP administration, which was attributed to the activity of released SMAC-P (Fig. 7g and S15). The IAP expression level of MSC-guided ApoLNP-administered tumors was 5.4-fold lower than those with saline treatment. The level was also 1.7-fold reduced to that of the IT administration, expecting that the MSC-guided ApoLNP administration would be highly effective in drug-resistant tumor species as well. Finally, it was fully confirmed that the MSC-guided administration of ApoLNPs could maximize the tumor-specific therapeutic efficacy, far reducing the off-target toxicity in normal organs.

## Discussion

One of the major problems of cancer therapy is the undesirable systemic distribution of anticancer drugs. The poor delivery efficiency of conventional anticancer drugs brings about insufficient therapeutic outcomes and the drugs transported to off-target tissues cause serious systemic toxicity. The toxic effects of drugs eventually limit their maximum dose, which in turn reduces their therapeutic effect again [52–54]. During the last four decades, nanoDDSs have been extensively explored to enhance the tumor-specific delivery of anticancer drugs. When administered intravenously, nanoDDSs were known to be accumulated specifically in tumor tissues via the EPR effect [55–57]. However, the anticancer efficacy of nanoDDSs was insufficient for effective cancer treatment, and other difficulties such as low drug capacity, carrier-associated toxicity, and formulation quality control were newly aroused by nanoDDSs [58–60]. It is consistently required to develop a new strategy for increasing the tumor-specific accumulation of anticancer drugs.

Herein, a new intratumoral drug delivery system comprised of an implantable MSC and drug-encapsulating LNPs was proposed as a method to promote the tumor-specific accumulation of anticancer drugs. MSC was precisely manufactured via the photolithography method, wherein all dimensions of its components including the entire size and thickness, needle length, and reservoir volume were freely adjustable considering the size of tumor tissues where MSC was to be administered. The diffusion rate of encapsulated LNPs from the reservoir to tumor tissues could be controlled depending on the inner width of the needle and the number of pinholes on the tip of the needle. The direct intratumoral administration of MSC was expected to relieve the undesirable drug distribution to normal tissues, and its steady drug infusion would diminish the leakage of drugs through blood and lymphatic vessels. In addition, an optimization study of LNP composition was carried out to obtain the most appropriate LNP for the enhanced and uniform drug distribution inside the tumor. The preferable surface property of LNPs for MSC-guided administration was different from those for intravenous administration since the LNPs were directly administered to tumors without undergoing systemic circulation. The PEGylated LNPs, a common method to prolong the circulation time and physiological stability of LNPs for their intravenous administration, was not advantageous for the MSC-guided administration because the steric hindrance by PEG impeded them from remaining inside the tumor tissues and being endocytosed by cancer cells [61]. Positively charged LNPs were not suitable either due to their low stability in the physiological condition and irregular intratumoral distribution. The LNP with slightly negative zeta potential (10 mol% of PS, 10PS) was rather found to be more favorable for MSC-guided drug delivery, demonstrating its high tumor accumulation level and uniform distribution throughout the whole tumor tissue. In addition, the volume and concentration of the 10PS solution loaded to the MSC were set to 1 μL and 30 mg/mL, respectively, to secure uniform intratumoral distribution of 10 PS while minimizing the extratumoral leakage of its excessive dose.

Cancer cell-specific and pro-apoptotic SMAC-P-FRRG-DOX was encapsulated in the LNPs with 10 mol% PS, resulting in ApoLNPs. The SMAC-P-FRRG-DOX prodrug was designed to be specifically cleaved to release SMAC-P and free DOX in response to cancer cell-overexpressed cathepsin B [46, 62, 63]. The cancer cell-specific activation of the prodrug was supposed to exhibit a synergistic effect with MSC-guided drug delivery, further greatly reducing the possible systemic toxicity that was usually induced by the toxic drug-loaded nanoDDS. Moreover, SMAC-P-FRRG-DOX contained two different therapeutic agents which enabled the combination therapy of chemodrug (DOX) and pro-apoptotic agent (SMAC-P). Although its IC_50_ value against 4T1 cancer cells was slightly higher than free DOX treatment due to its delayed activation by cathepsin B, it efficiently suppressed the anti-apoptotic reaction of the tumor by directly blocking IAPs. The combination therapy by ApoLNPs would not only facilitate overcoming the single chemotherapy-attributed drug resistance but also enhance the therapeutic effect of MSC-guided administration by compensating for the finite drug capacity of MSC.

When ApoLNPs were administered with the MSC guidance, it was localized inside the tumor at a much higher level compared to its intravenous administration, even at a far lower dose. In addition, the MSC-guided administration allowed ApoLNPs to remain inside tumor tissues for a prolonged time since the very slow infusion of ApoLNP through the MSC retarded its fast intratumoral clearance by lymphatic drainage. ApoLNPs showed much prolonged intratumoral retention compared to free DOXs and SMAC-P-FRRG-DOXs in their MSC-guided intratumoral administration, confirming the advantage of LNP encapsulation in intratumoral drug diffusivity control and prevention of its rapid extratumoral washout. The improved tumor accumulation of MSC-guided administered ApoLNPs directly resulted in its enhanced therapeutic efficacy as it exhibited exceptional tumor growth inhibition compared to the intratumorally administered one without MSC. The expression of IAPs inside tumor tissue was also significantly depressed, validating the pro-apoptotic property of ApoLNPs in vivo. Notably, the cooperative effect of the low dose of ApoLNPs, tumor-exclusive administration by MSC guidance, and the cathepsin B-specific activation of the prodrug significantly lessened the systemic toxicity. Consequently, the proposed MSC-guided LNP administration system was confirmed to be a potent platform technique to modulate the delivery efficiency of therapeutic agents. So far, the application area of the MSC-guided intratumoral LNP administration system is restricted to the treatment of primary solid tumors and it is hard to draw out therapeutic effects on secondary, distant, or metastatic tumors. The limitation of MSC-guided intratumoral LNP administration is expected to be overcome by its utilization in cancer immunotherapy. Intratumoral administration is one of the major routes frequently adopted for cancer immunotherapy since the tumor-specific delivery of immunotherapeutic agents is essential for adequate anticancer immune activation and prevention of undesirable immunotoxicity [64–66]. In addition, multiple immunotherapeutic agents should be simultaneously administered at a controlled ratio to defeat the immunosuppressive tumor microenvironments and maximize the anticancer immune responses [67, 68]. MSC-guided intratumoral administration is considered highly beneficial for cancer immunotherapy since it would facilitate tumor-exclusive localization and prolonged retention of multiple immunotherapeutic agents at their precise dose ratios, enhancing the immunotherapeutic efficacy [69]. Moreover, sufficient anticancer immune activation inside the primary tumors by the MSC-guided administration would lead to the suppression of distant and metastatic tumor growth, which can effectively solve the problem of MSC-guided intratumoral administration. In the following studies, the application of MSC-guided intratumoral LNP administration to cancer immunotherapy will be conducted further.

## Conclusion

In this study, an MSC-based sustained-releasing drug delivery system was newly designed for the tumor-exclusive delivery of anticancer nanoDDS. The favorable LNP composition that can promote sufficient intratumoral drug infusion and distribution was proposed for the MSC-guided administration. The utilization of LNP encapsulation was confirmed to be advantageous in achieving prolonged tumor retention and suppressing extratumoral leakage, as compared with the MSC-guided administration of free drugs. Cancer-specific and pro-apoptotic SMAC-P-FRRG-DOX prodrugs were encapsulated into LNPs to form ApoLNPs, and the ApoLNPs were proven with their s pro-apoptotic property and cathepsin B specificity in cancer cells. When ApoLNPs were administered through MSC, their tumor accumulation was notably increased whereas its off-target delivery was significantly reduced compared to intravenous or intratumoral administration. Finally, the therapeutic efficacy of MSC-guided ApoLNP administration was confirmed in vivo, showing the strict inhibition of IAPs expression as well as tumor growth. The MSC-guided intratumoral LNP administration was expected to maximize the therapeutic efficacy of conventional nanoDDSs by overcoming the low delivery efficiency at targeted tumor tissues. In recent studies on cancer immunotherapy, intratumoral administration is being frequently adopted more and more since it was discovered that combination therapy with the cancer-specific and simultaneous delivery of two or more immunotherapeutic agents is essential for sufficient stimulation of anticancer immune responses. The MSC-guided intratumoral LNP administration can be also a potential platform strategy in intratumoral immunotherapy as it delivers multiple immunotherapeutic agents exclusively into the tumor tissues at a precise ratio, thereby possibly enhancing the immune activation efficacy.

### Supplementary Information

**Additional file 1:** **Fig. S1.** Schematic illustration describing the fabrication process of the implantable MSC for targeted anticancer therapy. **Fig. S2.** Time-dependent changes in sizes of LNPs (1 mg/mL) with an addition of 10% FBS, analyzed *via* DLS. **Fig. S3.** Digital images showing the MSC protection cap. **Fig. S4.** (a) IVIS images before and after the administration of MSCs charged with six LNPs. (b) The release rates of LNPs from MSCs, calculated as the ratio of fluorescence intensities before and after the administration. **Fig. S5. **(a) The HPLC and (b) LC-MS spectra of DOX and synthesized SMAC-P-FRRG-DOX (m/z = 841.7, [M]/2 + 1H^+^ and 1683.2, [M] + 2H^+^). **Fig. S6. **LC-MS spectra of G-DOX cleaved from SMAC-P-FRRG-DOX after being incubated with 10 μg/mL cathepsin B for 24 h (m/z = 623.3, [M] + Na^+^). **Fig. S7. ***Ex vivo *CLSM images of whole tumor sections after the saline treatment. **Fig. S8.** The quantification data showing relative fluorescence intensities of ApoLNPs over DAPI inside the whole tumor sections depending on their administration routes, analyzed *via* the CLSM (*n* =3). ns = not significant, *** *p* < 0.001, **** *p* < 0.0001. **Fig. S9.** (a) IVIS images of tumor tissues treated with free DOXs, SMAC-P-FRRG-DOXs, and ApoLNPs via MSC-guided intratumoral administration, and **(b)** their fluorescence intensity quantification over 48 h. **Fig. S10.** The *ex vivo* fluorescence intensities of normal organs after the treatment of ApoLNPs *via* different administration routes, showing the biodistribution of ApoLNPs over 48 h. **Fig. S11. **IVIS images of mice repeatedly treated with ApoLNPs (0.15 mg/kg, based on DOX content) through intratumoral injection without MSC guidance (three times at 0, 72, and 144 h). **Fig. S12.** (a) *Ex vivo* IVIS images of normal organs collected from mice receiving three shots of ApoLNPs by intratumoral or MSC-guided administration (0.15 mg/kg, based on DOX content, once per three days), and (b) their quantification data. **Fig. S13.** Body weight changes of recipient mice during 12 days of ApoLNP treatment through different routes. **Fig. S14.** The H&E histology analysis of normal organs upon finishing the 12-day ApoLNP treatment with different administration routes. **Fig. S15.** The western blot assay of tumor tissues treated with ApoLNPs *via* different administration routes to compare their IAP expression levels. 

## Data Availability

All data obtained throughout this study are presented in the manuscript or supporting information.
