# Supplementary material for: Micro-syringe chip-guided intratumoral administration of lipid nanoparticles for targeted anticancer therapy
Source: Biomater Res. 2023 Oct 16;27:102. doi: 10.1186/s40824-023-00440-4 (PMC10577945; doi:10.1186/s40824-023-00440-4)
Supplement: Supplementary file 1 — Additional file 1: Fig. S1. Schematic illustration describing the fabrication process of the implantable MSC for targeted anticancer therapy. Fig. S2. Time-dependent changes in sizes of LNPs (1 mg/mL) with an addition of 10% FBS, analyzed via DLS. Fig. S3. Digital images showing the MSC protection cap. Fig. S4. (a) IVIS images before and after the administration of MSCs charged with six LNPs. (b) The release rates of LNPs from MSCs, calculated as the ratio of fluorescence intensities before and after the administration. Fig. S5. (a) The HPLC and (b) LC-MS spectra of DOX and synthesized SMAC-P-FRRG-DOX (m/z = 841.7, [M]/2 + 1H+ and 1683.2, [M] + 2H+). Fig. S6. LC-MS spectra of G-DOX cleaved from SMAC-P-FRRG-DOX after being incubated with 10 μg/mL cathepsin B for 24 h (m/z = 623.3, [M] + Na+). Fig. S7. Ex vivo CLSM images of whole tumor sections after the saline treatment. Fig. S8. The quantification data showing relative fluorescence intensities of ApoLNPs over DAPI inside the whole tumor sections depending on their administration routes, analyzed via the CLSM (n =3). ns = not significant, *** p < 0.001, **** p < 0.0001. Fig. S9. (a) IVIS images of tumor tissues treated with free DOXs, SMAC-P-FRRG-DOXs, and ApoLNPs via MSC-guided intratumoral administration, and (b) their fluorescence intensity quantification over 48 h. Fig. S10. The ex vivo fluorescence intensities of normal organs after the treatment of ApoLNPs via different administration routes, showing the biodistribution of ApoLNPs over 48 h. Fig. S11. IVIS images of mice repeatedly treated with ApoLNPs (0.15 mg/kg, based on DOX content) through intratumoral injection without MSC guidance (three times at 0, 72, and 144 h). Fig. S12. (a) Ex vivo IVIS images of normal organs collected from mice receiving three shots of ApoLNPs by intratumoral or MSC-guided administration (0.15 mg/kg, based on DOX content, once per three days), and (b) their quantification data. Fig. S13. Body weight changes of recipient mice duri [file 40824_2023_440_MOESM1_ESM.docx]

**Supplementary Information for**

Micro-syringe chip-guided intratumoral administration of lipid nanoparticles for targeted anticancer therapy

Jeongrae Kim^1,2†^, Sunejeong Song^1†^, Minjun Gwak^3†^, Hanhee Cho^1^, Wan Su Yun^1,2^, Namcheol Hwang^4^, Jinseong Kim^1,2^, Jun Seo Lee^3^, Dong-Hwee Kim^2^, Hyuncheol Kim^4^, Seong Ik Jeon^1*^, Tae-il Kim^3*^, Kwangmeyung Kim^1*^

^1^College of Pharmacy, Graduate School of Pharmaceutical Sciences, Ewha Woman’s University, Seoul 03760, Republic of Korea.

^2^KU-KIST Graduate School of Converging Science and Technology, Korea University, Seoul, 02841, Republic of Korea.

^3^School of Chemical Engineering, Sungkyunkwan University (SKKU), Suwon 16419, Republic of Korea.

^4^Department of Chemical and Biomolecular Engineering, Sogang University, Seoul 04107, Republic of Korea.

^†^These authors contributed equally to this work.

*Correspondence

Seong Ik Jeon (jeonseongik@gmail.com), Kwangmeyung Kim (kimkm@ewha.ac.kr)

College of Pharmacy, Graduate School of Pharmaceutical Sciences, Ewha Woman’s University, Seoul 03760, Republic of Korea.

Tae-il Kim (taeilkim@skku.edu)

School of Chemical Engineering, Sungkyunkwan University (SKKU), Suwon 16419, Republic of Korea.

**
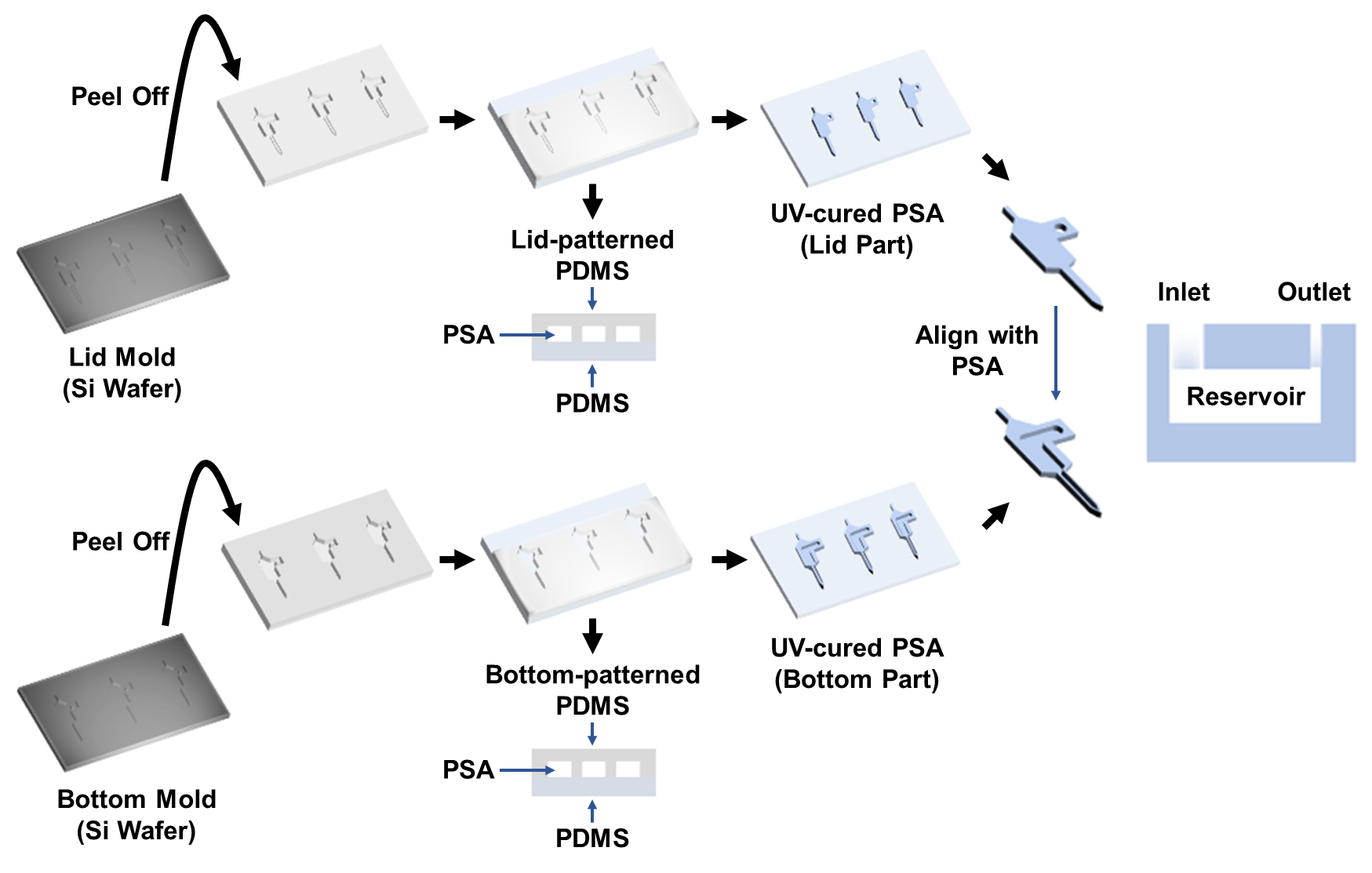
**

**Fig. S1.** Schematic illustration describing the fabrication process of the implantable MSC for targeted anticancer therapy.

**Fig. S2.** Time-dependent changes in sizes of LNPs (1 mg/mL) with an addition of 10% FBS, analyzed *via* DLS.

**
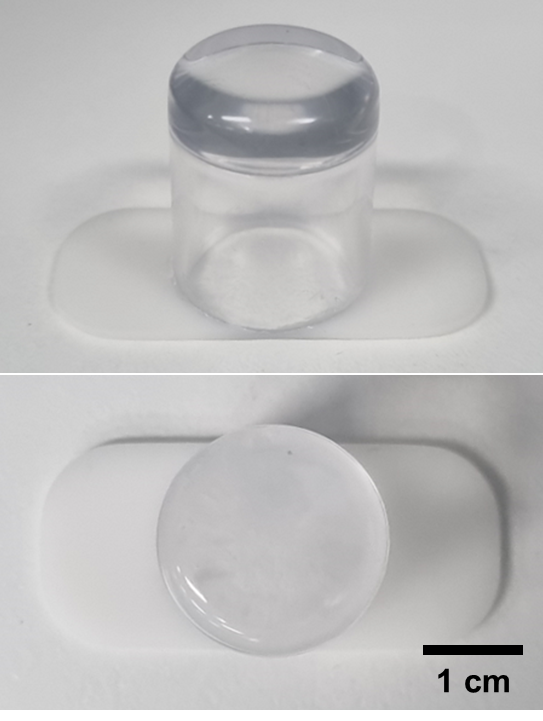
**

**Fig. S3.** Digital images showing the MSC protection cap.


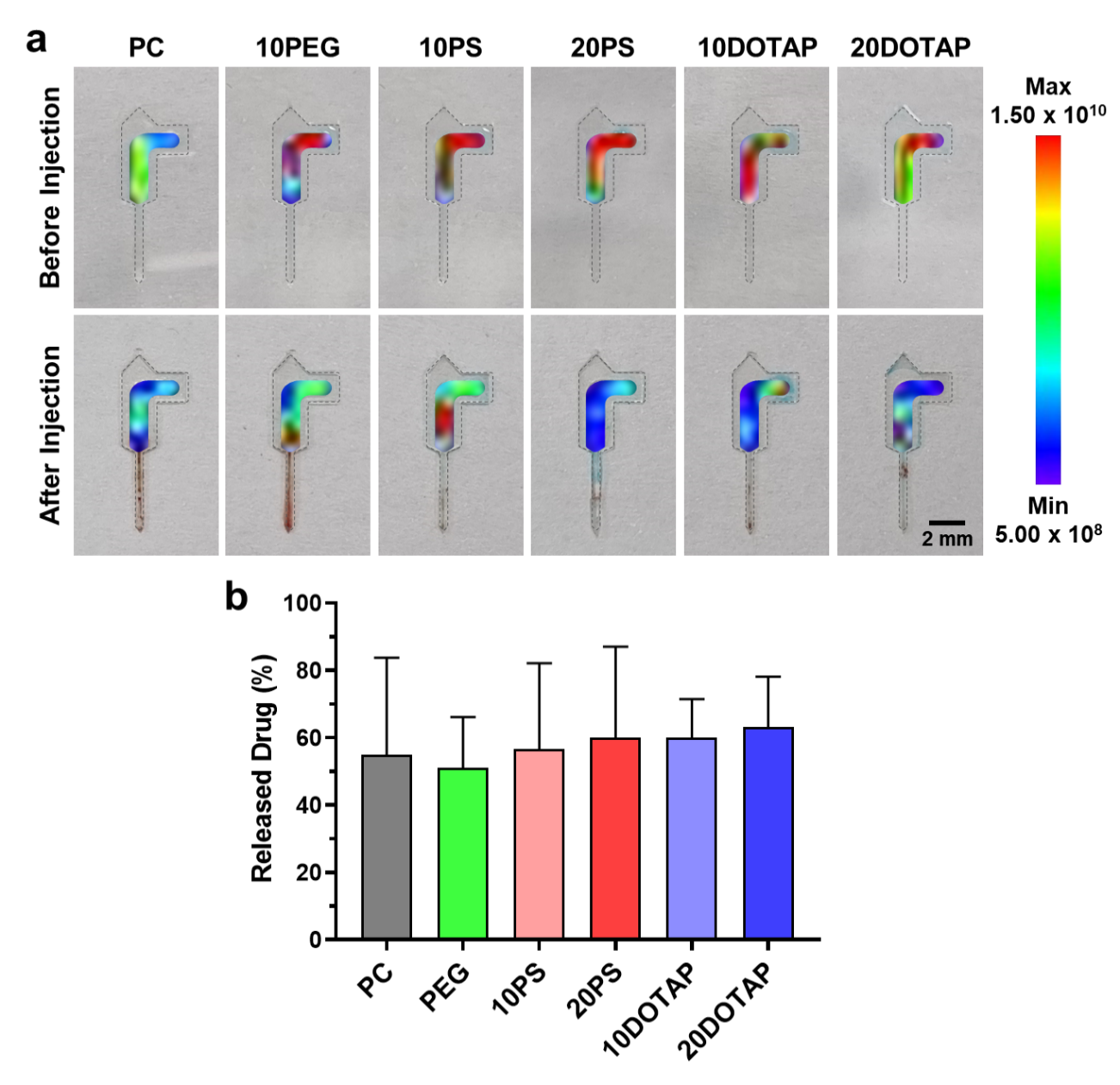


**Fig. S4.** **(a)** IVIS images before and after the administration of MSCs charged with six LNPs. **(b)** The release rates of LNPs from MSCs, calculated as the ratio of fluorescence intensities before and after the administration.


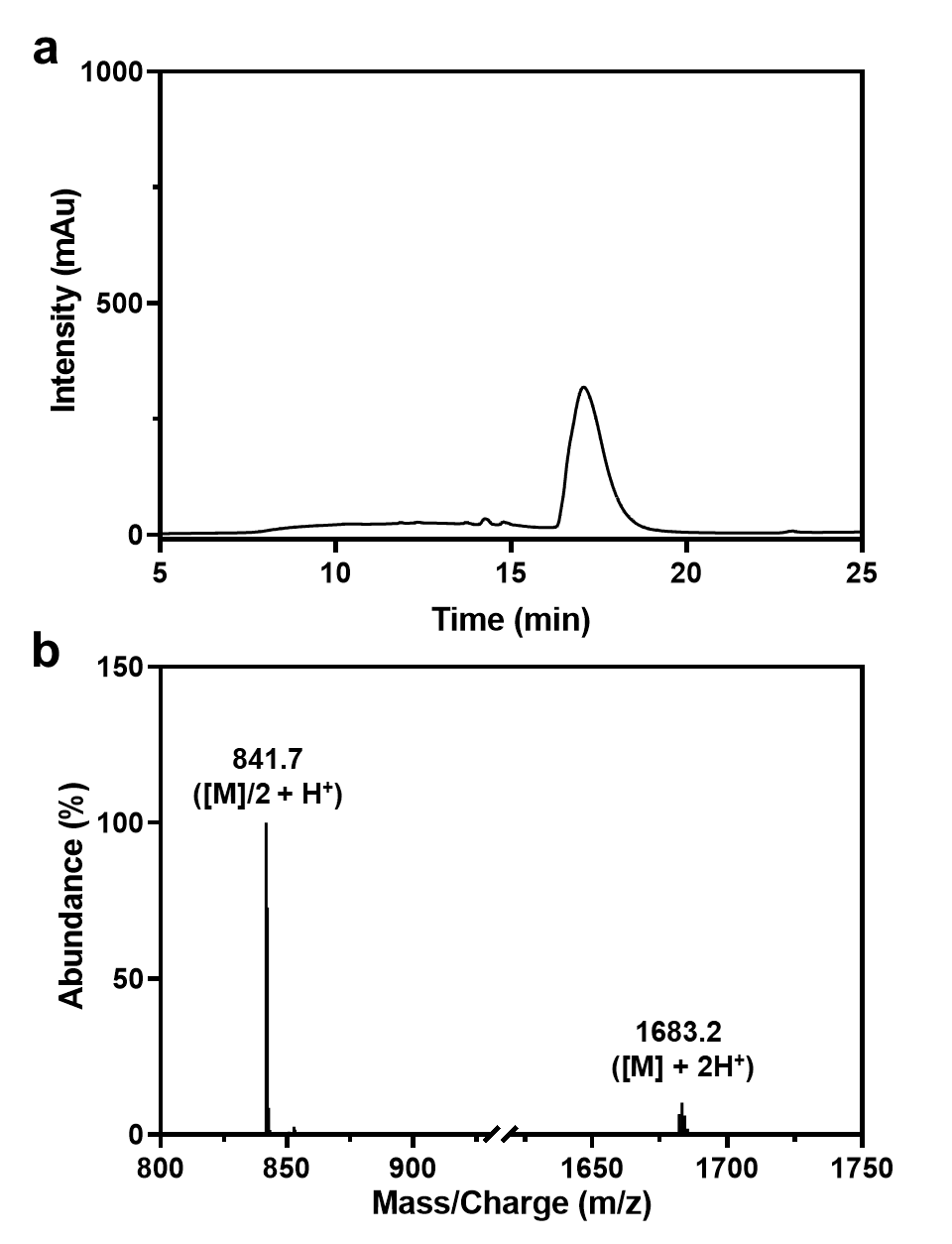


**Fig. S5. (a)** The HPLC and **(b)** LC-MS spectra of DOX and synthesized SMAC-P-FRRG-DOX (m/z = 841.7, [M]/2 + 1H^+^ and 1683.2, [M] + 2H^+^).


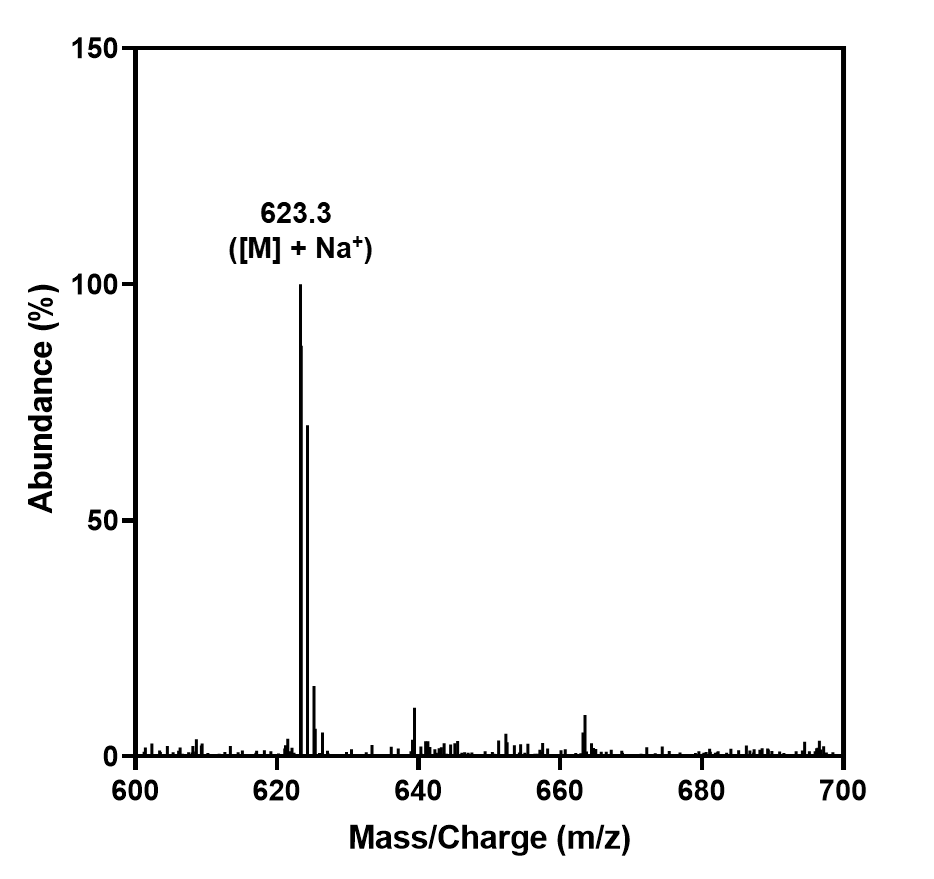


**Fig. S6.** LC-MS spectra of G-DOX cleaved from SMAC-P-FRRG-DOX after being incubated with 10 μg/mL cathepsin B for 24 h (m/z = 623.3, [M] + Na^+^).


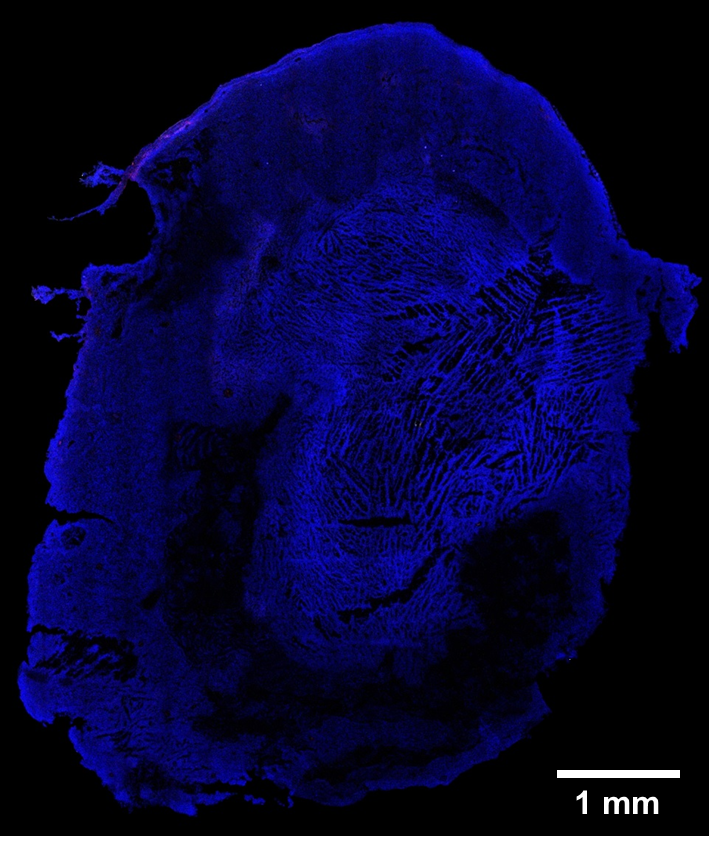


**Fig. S7.** *Ex vivo* CLSM images of whole tumor sections after the saline treatment.

**Fig. S8.** The quantification data showing relative fluorescence intensities of ApoLNPs over DAPI inside the whole tumor sections depending on their administration routes, analyzed *via* the CLSM (n =3). ns = not significant, *** p < 0.001, **** p < 0.0001.


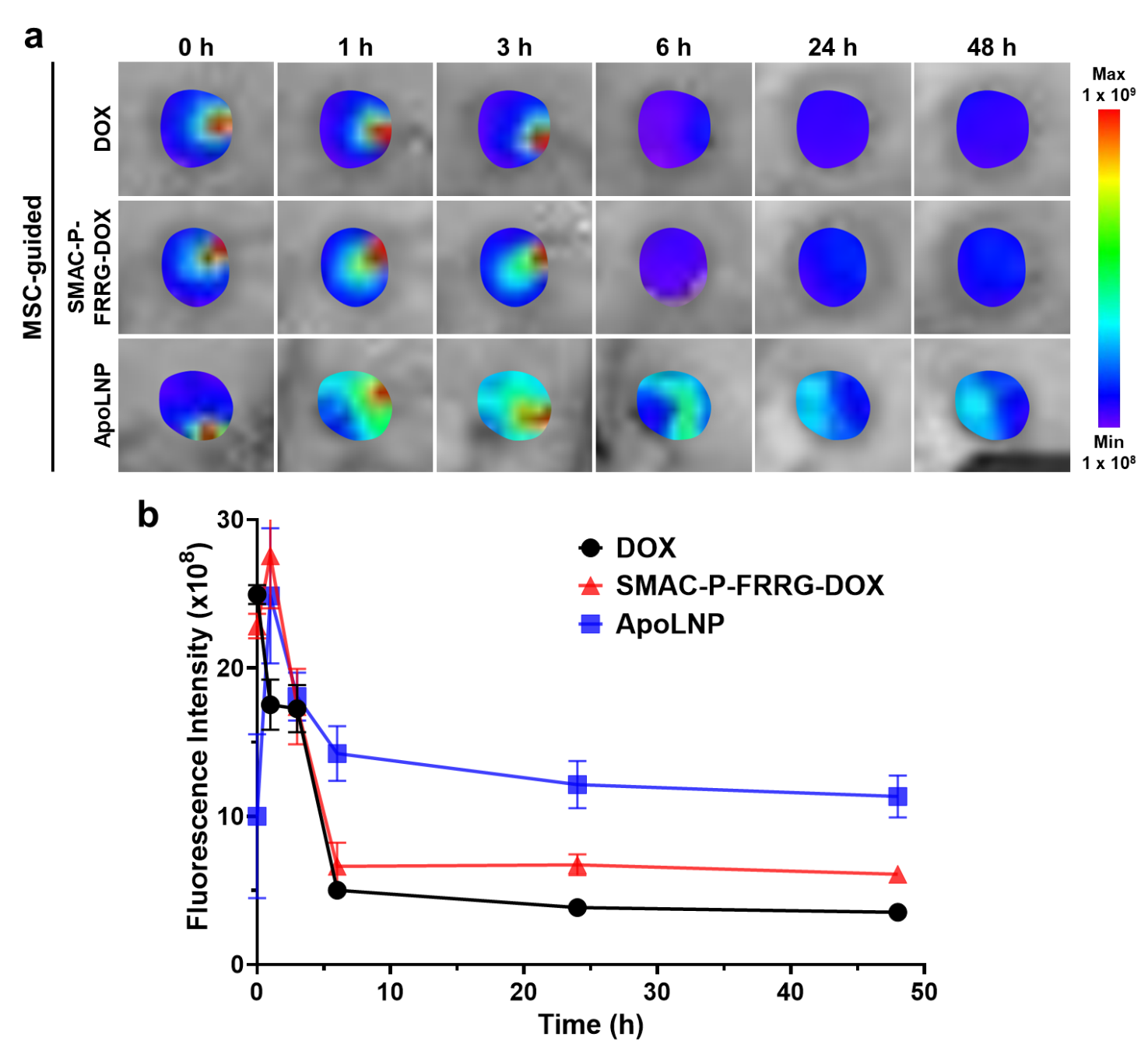


**Fig. S9. (a)** IVIS images of tumor tissues treated with free DOXs, SMAC-P-FRRG-DOXs, and ApoLNPs via MSC-guided intratumoral administration, and **(b)** their fluorescence intensity quantification over 48 h.


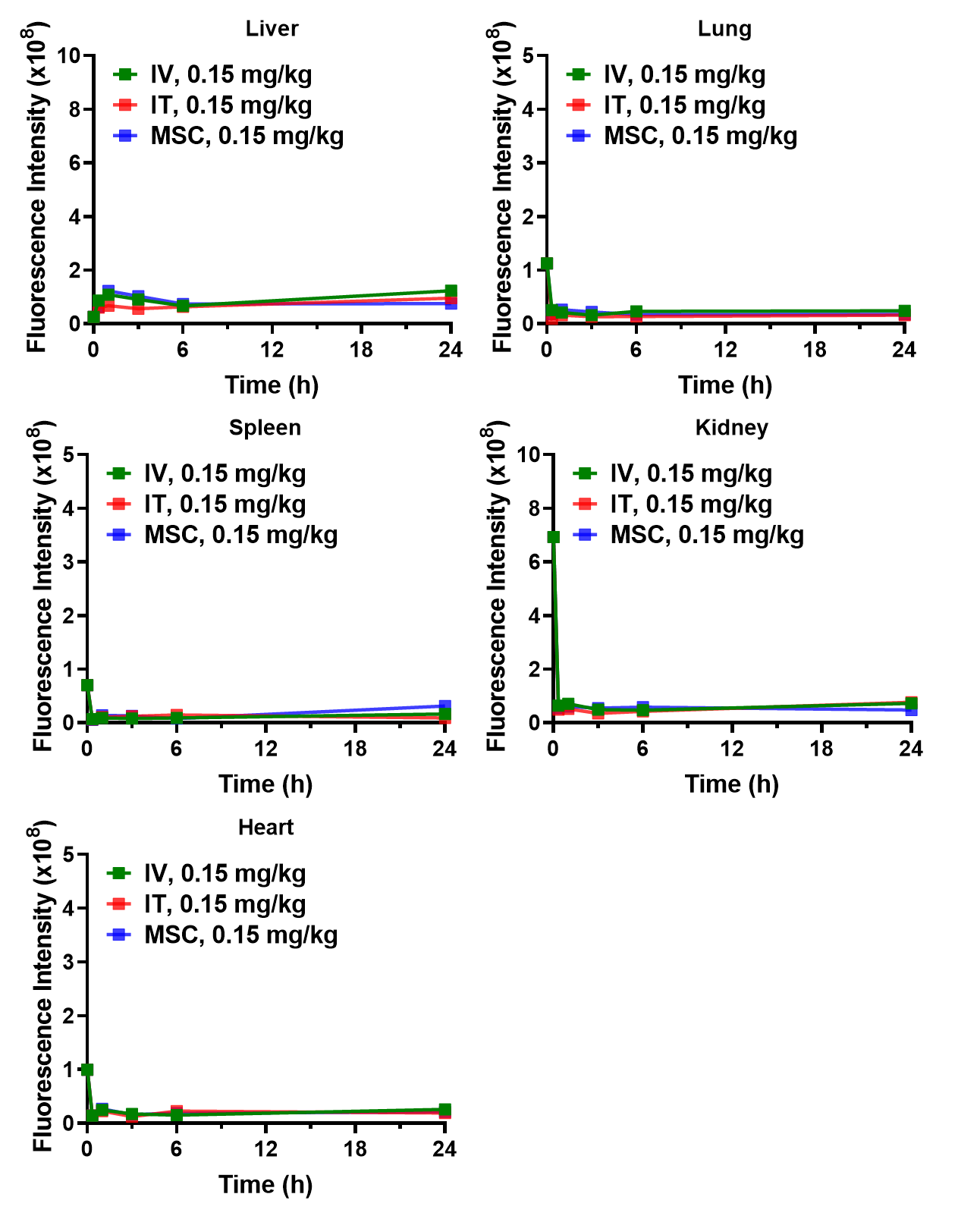


**Fig. S10.** The *ex vivo* fluorescence intensities of normal organs after the treatment of ApoLNPs *via* different administration routes, showing the biodistribution of ApoLNPs over 48 h.


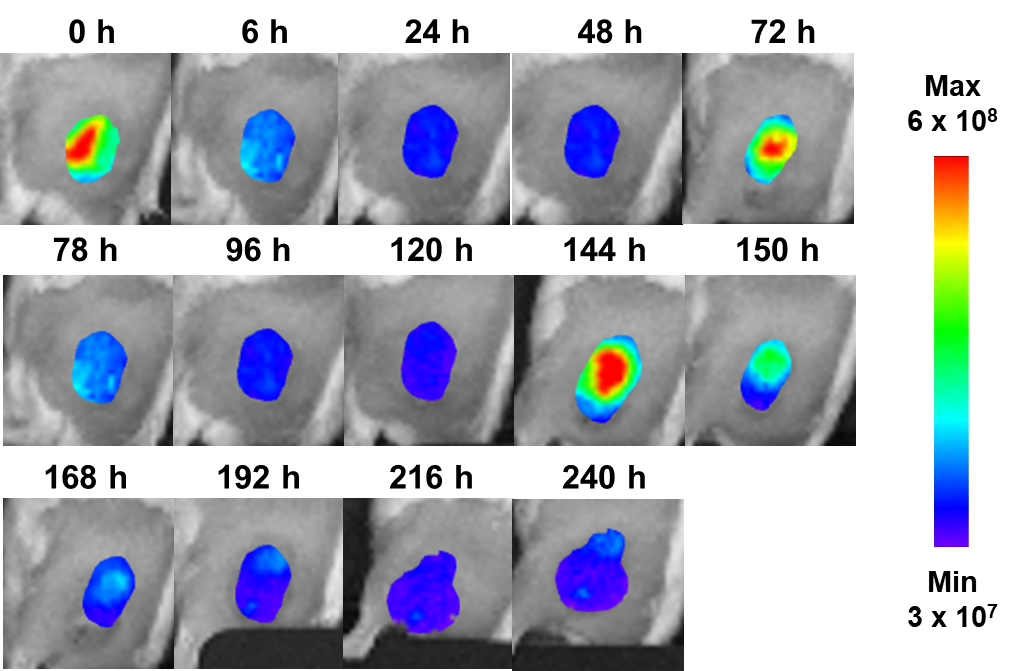


**Fig. S11.** IVIS images of mice repeatedly treated with ApoLNPs (0.15 mg/kg, based on DOX content) through intratumoral injection without MSC guidance (three times at 0, 72, and 144 h).

**
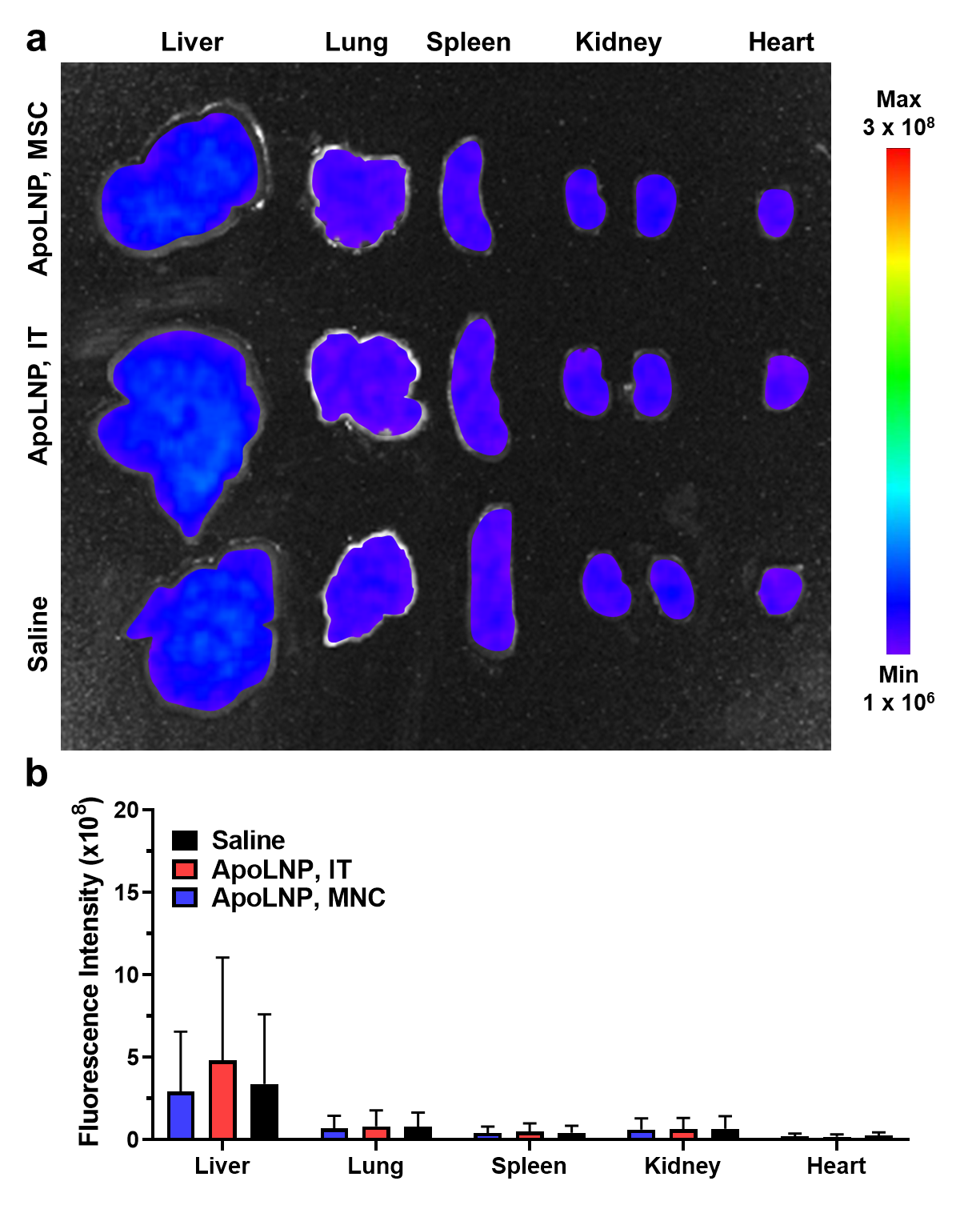
**

**Fig. S12.** **(a)** *Ex vivo* IVIS images of normal organs collected from mice receiving three shots of ApoLNPs by intratumoral or MSC-guided administration (0.15 mg/kg, based on DOX content, once per three days), and **(b)** their quantification data.

**Fig. S13.** Body weight changes of recipient mice during 12 days of ApoLNP treatment through different routes.


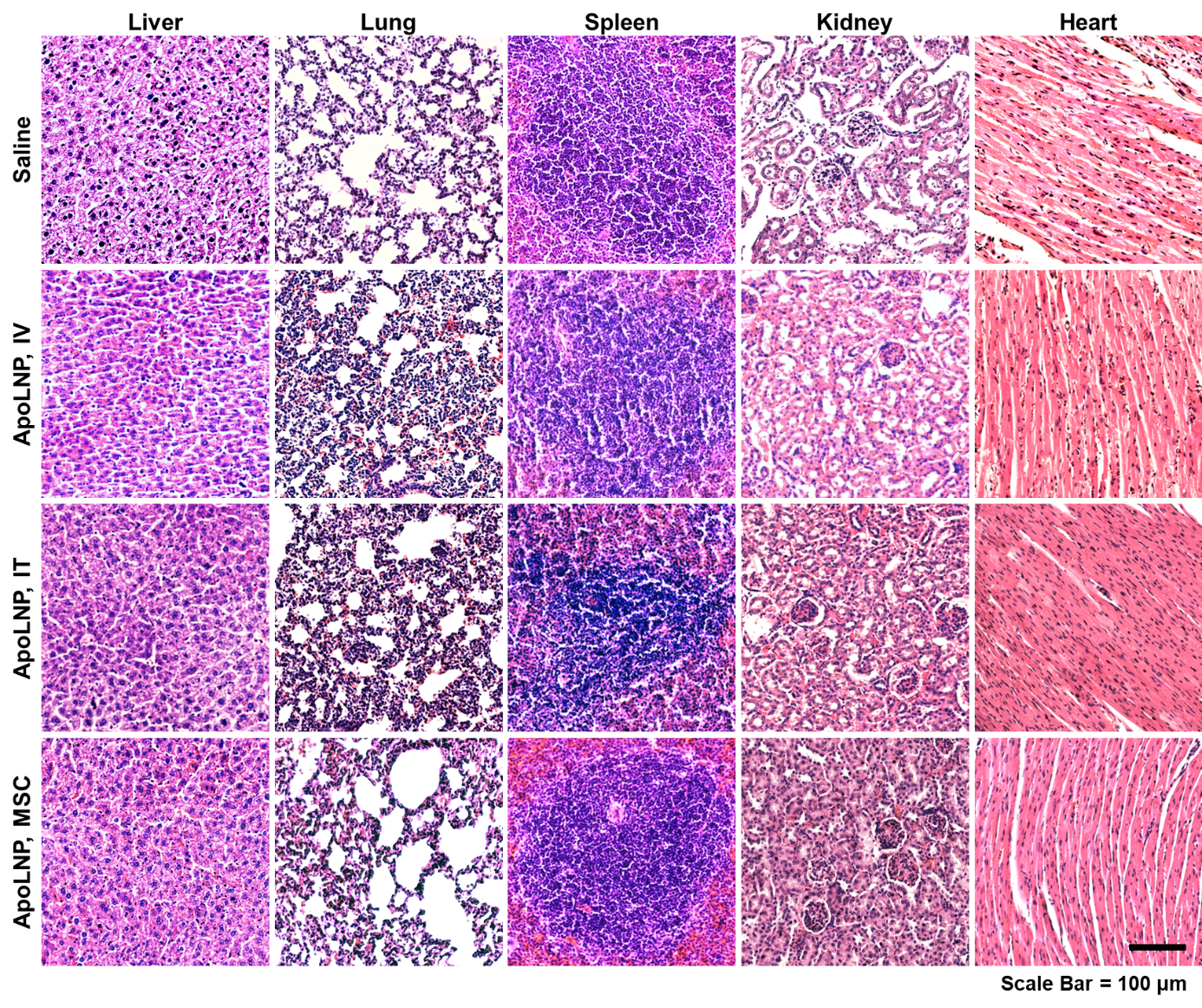


**Fig. S14.** The H&E histology analysis of normal organs upon finishing the 12-day ApoLNP treatment with different administration routes.


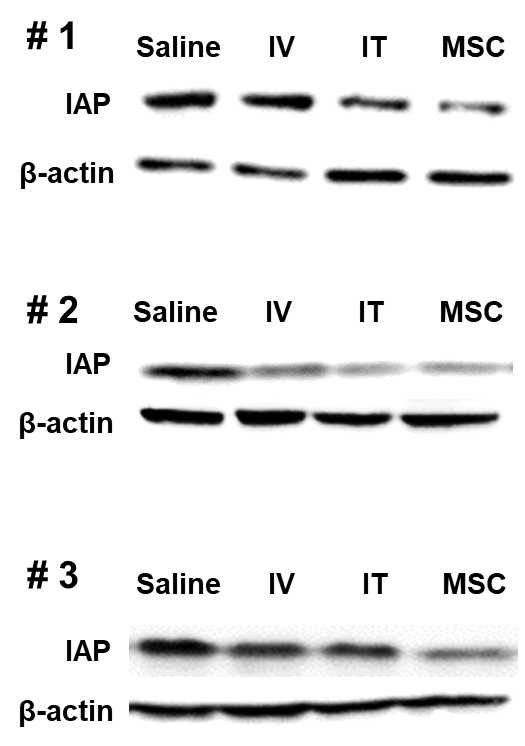


**Fig. S15.** The western blot assay of tumor tissues treated with ApoLNPs *via* different administration routes to compare their IAP expression levels.
